# Role of RNA structural plasticity in modulating HIV-1 genome packaging and translation

**DOI:** 10.1073/pnas.2407400121

**Published:** 2024-08-07

**Authors:** Saif Yasin, Sydney L. Lesko, Siarhei Kharytonchyk, Joshua D. Brown, Issac Chaudry, Samuel A. Geleta, Ndeh F. Tadzong, Mei Y. Zheng, Heer B. Patel, Gabriel Kengni, Emma Neubert, Jeanelle Mae C. Quiambao, Ghazal Becker, Frances Grace Ghinger, Sreeyasha Thapa, A’Lyssa Williams, Michelle H. Radov, Kellie X. Boehlert, Nele M. Hollmann, Karndeep Singh, James W. Bruce, Jan Marchant, Alice Telesnitsky, Nathan M. Sherer, Michael F. Summers

**Affiliations:** ^a^Department of Chemistry and Biochemistry, University of Maryland, Baltimore County, MD 21250; ^b^Department of Oncology, McArdle Laboratory for Cancer Research, University of Wisconsin-Madison, Madison, WI 53705; ^c^Department of Oncology, Institute for Molecular Virology, University of Wisconsin-Madison, Madison, WI 53705; ^d^Department of Microbiology and Immunology, University of Michigan Medical School, Ann Arbor, MI 48109-5620; ^e^HHMI, University of Maryland, Baltimore County, MD 21250; ^f^Department of Chemistry and Biochemistry, University of Maryland, Baltimore, MD 21250

**Keywords:** HIV-1, polyA, dimerization, translation, packaging

## Abstract

HIV-1 transcript function is controlled in part by twinned transcriptional start site usage, where 5′ capped RNAs beginning with a single guanosine (1G) are preferentially packaged into progeny virions as genomic RNA (gRNA) whereas those beginning with three sequential guanosines (3G) are retained in cells as mRNAs. In 3G transcripts, one of the additional guanosines base pairs with a cytosine located within a conserved 5′ polyA element, resulting in formation of an extended 5′ polyA structure as opposed to the hairpin structure formed in 1G RNAs. To understand how this remodeling influences overall transcript function, we applied in vitro biophysical studies with in-cell genome packaging and competitive translation assays to native and 5′ polyA mutant transcripts generated with promoters that differentially produce 1G or 3G RNAs. We identified mutations that stabilize the 5′ polyA hairpin structure in 3G RNAs, which promote RNA dimerization and Gag binding without sequestering the 5′ cap. None of these 3G transcripts were competitively packaged, confirming that cap exposure is a dominant negative determinant of viral genome packaging. For all RNAs examined, conformations that favored 5′ cap exposure were both poorly packaged and more efficiently translated than those that favored 5′ cap sequestration. We propose that structural plasticity of 5′ polyA and other conserved RNA elements place the 5′ leader on a thermodynamic tipping point for low-energetic (~3 kcal/mol) control of global transcript structure and function.

## Significance

HIV-1 utilizes a twinned transcriptional start site (TTSS) mechanism to expand the function of its integrated DNA provirus. The present study reveals how RNA structural plasticity within the 5′ leader of HIV-1 transcripts enables TTSS-dependent exposure of RNA elements that differentially promote genome versus mRNA functions. We show that the propensity for 5′ cap exposure is dominant over RNA dimerization and Gag binding in controlling genome packaging and translation activity, and that structural plasticity of a conserved 5′ RNA element (5′ polyA) is critical for enabling TTSS control of transcript function.

**Fig. 1.**
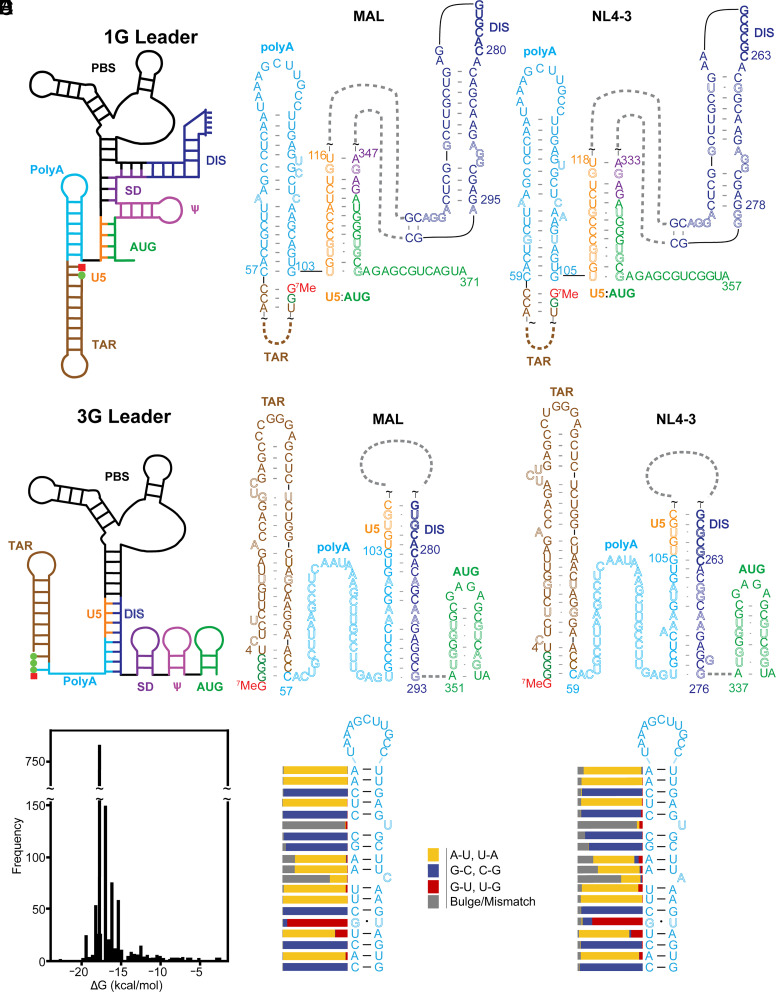
Conserved HIV-1 5′ polyA element forms hairpin and extended structures in 1G (*A*) and 3G (*B*) 5′ leader RNAs, respectively. (*A* and *B*) Folding cartoon (*Left*) and regions of NMR-derived secondary structures of 1G (*A*) and 3G (*B*) of HIV-1_MAL_ and HIV-1_NL4-3_ leader RNAs. Base pairings between U5 and DIS of the HIV-1_NL4-3_ leader were detected by NMR (18) and the DIS-polyA base pairs were inferred from the MAL structure (9). Residue colors denote different secondary structure elements; noncanonical base pairs and bulges are shown in outlined fonts; green dots and red squares denote guanosines and 5' cap residues, respectively. (*C*) Histogram of 5′ polyA hairpin free energies showing a narrow distribution about −17.0 kcal/mol. (*D*) Consensus 5′ polyA secondary structure identified by locARNA (19–21) showing the locations of conserved bulges across all 5′ polyA hairpins identified. (*E*) Consensus secondary structure and conserved bulge locations for the 186 unique 5′ polyA hairpins identified. Bars denote % conservation at each position.

Retroviruses utilize diverse mechanisms to expand the functional output of their single integrated DNA provirus, including alternative splicing to produce viral accessory proteins and the envelope protein, frameshifting during translation to produce the Gag and Gag-Pol polyproteins, and proteolytic cleavage of Gag and Gag-Pol during viral maturation to produce the mature proteins of infectious virions (1). An additional diversification mechanism was recently uncovered that involves the production of RNAs that differ at their 5′ ends due to twinned transcriptional start site (TTSS) usage (2). RNAs transcribed with a single 5′ guanosine (1G) are packaged into progeny virions where they function as genomic RNA (gRNA), whereas those transcribed with three sequential guanosines (3G) are retained in cells and function as mRNAs (2–8). NMR (NMR) and chemical probing studies revealed that 1G and 3G RNAs adopt dramatically different structures (9, 10) (Fig. 1 *A*–*B*). 5′-capped 1G RNAs adopt a branched multihairpin structure in which elements important for transcription initiation, reverse transcription, dimerization, and packaging (TAR, PBS, DIS, and ^HP^, respectively) exist as hairpins that decorate a central tandem three-way junction. The dimeric leader exposes ~20 high-affinity binding sites for the cognate nucleocapsid (NC) domains of the viral Gag protein (11, 12), supporting the hypothesis that dimerization-dependent Gag binding is a requirement for genome packaging (13–17). In contrast, 5′-capped 3G RNAs are remodeled such that residues important for dimerization and NC binding are sequestered, whereas those believed to be important for splicing and translation are exposed (9). Additional studies revealed that the 5′ cap is sequestered and inaccessible to cap-binding proteins in the 1G transcript, but exposed and accessible in 3G RNAs, suggesting that TTSS-modulated 5′ cap exposure may also play a role in controlling gRNA versus mRNA function (3, 8, 9).

The full-length HIV-1 transcript contains two copies of a “repeat sequence” (R), one located within the 5′ leader and the other at the 3′ end of the RNA (22, 23), both of which include a ~47 nucleotide element containing a polyadenylation signal called polyA. The function of the 3′ polyA element is well understood: It recruits the cleavage polyadenylation specificity factor (CPSF) and other factors that together initiate 3′ polyadenylation. Comparatively less is known about the function of the 5′ polyA element, which lacks an upstream sequence element (USE) and auxiliary downstream sequence element required to promote polyadenylation (24–26). Secondary structure predictions and chemical probing studies suggested that the 5′ polyA element forms a hairpin that contains multiple bulges or mismatched residues within its stem (27). Previous studies suggested that the conserved bulges may serve as binding sites for polyadenylation factors (28, 29), or the viral accessory protein, Vif (30). The 5′ polyA bulges were also implicated in Gag binding (28, 29), although studies using mutational interference mapping (MIME), photoactivatable ribonucleoside-enhanced crosslinking and immunoprecipitation (PAR-CLIP) (31, 32), and calorimetric titration studies (33) suggest that the 5′ polyA element does not exhibit significant affinity for Gag or its NC domain. Importantly, mutations engineered to either increase or decrease the stability of the 5′ polyA hairpin substantially attenuated viral replication (27, 28, 34), and viruses with mutated 5′ polyA hairpins that possessed altered hairpin stabilities evolved revertant mutations with wild-type stabilities (35). These studies revealed that viral infectivity is dependent on delicately balanced 5′ polyA structural stability.

Evolution of a 5′ polyA hairpin with tuned stability could enable repression of 5′ polyadenylation while simultaneously promoting 3′ polyadenylation (36). More recent studies suggest 5′ polyA stability may also be tuned to facilitate formation of multiple 5′ leader structures with distinct functions. NMR and chemical probing studies have shown that the 5′ polyA element in both capped and uncapped 5′ leader constructs adopts distinct, dimerization-dependent structures, forming a hairpin in the dimeric RNA and an extended structure in the monomer (Fig. 1 *A* and *B*) (although details of the secondary structures differ; *SI Appendix*, Fig. S1) (9, 10, 17, 37). NMR studies revealed that the additional base pair between the 5′ guanosine of the 3G leader and the 5′ cytosine of the 5′ polyA element is the primary trigger for TTSS-dependent structural changes (2, 9).

To understand how these structural changes induce differences in transcript function, we have now combined a phylogenetic analysis with in vitro and in cell studies of 5′-capped leader transcripts containing native and mutated 5′ polyA elements with stabilized-hairpin structures. We also developed an in-cell competitive translation assay that enables direct measurement of translation efficiencies for 1G and 3G transcripts. Our studies focused on both the laboratory-adapted NL4-3 strain of HIV-1 (subtype B; HIV-1_NL4-3_) and the MAL strain that is widely distributed among humans (M group subtype A; HIV-1_MAL_). Our findings suggest that HIV-1 evolved a 5′ polyA sequence tuned for structural plasticity, enabling TTSS-modulated control of packaging versus translation functions. This work sheds light on the relative contributions of RNA dimerization, exposure of Gag binding sites, and 5′ cap exposure to viral RNA transcript form and function.

## Results

### Evolutionarily Conserved Bulges Tune the Stability of the 5′ polyA Hairpin.

NMR studies have shown that the 5′ polyA region of HIV-1_MAL_ forms a hairpin in the capped 1G 5′ leader and an extended structure in the capped 3G leader (9). Similar structures (but all with different base pairings in the monomeric conformation; *SI Appendix*, Fig. S1) have been proposed from chemical probing experiments of uncapped RNAs from HIV-1_NL4-3_ and HIV-1_LAI_ (10, 17, 37, 38). In all cases, the stem of the 5′ polyA hairpin in the dimeric RNA includes multiple mismatched and bulged residues expected to reduce the stability of the hairpin. A bioinformatics analysis found that the free energy of the 5′ polyA hairpin is finely tuned across a small subset of HIV-1 strains (27), and a more extensive study found that two-thirds of 1,863 HIV-1 isolates examined had a predicted free energy of −18.5 kcal/mol (39), which is much higher than the free energy expected for a hairpin containing only canonical base pairs (−27.1 kcal/mol, Table 1). To determine whether conserved structural features regulate 5′ polyA hairpin stability, we generated a consensus secondary structure across all isolate sequences deposited in the Los Alamos National Laboratory HIV sequence compendium (40). To locate 5′ polyA hairpins, we first searched for the polyadenylation signal (5′-AAUAAA-3′) (41) within each 5′ leader deposition and then identified the hairpin exhibiting the lowest predicted free energy that includes the signal (*SI Appendix*, Fig. S2*A*) (Supplemental Information for details). A total of 186 unique 5′ polyA sequences were found within the 1,268 total 5′ polyA hairpins identified, with the majority being derived from subtype B isolates (*SI Appendix*, Fig. S2*B*).

**Table 1.** Predicted free energies of secondary structure elements of the HIV-1_MAL_ 5′ and HIV-1_NL4-3_ 5′ leaders^*^

	PolyA Hairpin	DIS Hairpin	AUG Hairpin	U5:AUG Helix	U5:DIS Helix
Average of Representative Strains^†^	−16.7 ± 1.6	−13.8 ± 1.4	−3.3 ± 0.5	−16.3 ± 1.3	−12.7 ± 4.2
MAL	−15.7	−13.0	−3.1	−17.0	−19.6
Idealized MAL	−27.1	−27.7	−17.4	−25.4	−31.6
ΔΔG	11.4	14.7	14.3	8.4	16.5
NL4-3	−15.4	−13.2	−4.7	−16.6	−13.3
Idealized NL43	−27.1	−27.7	−17.3	−26.6	−34.6
ΔΔG	11.7	14.5	12.6	10.0	21.3

^*^Values in kcal/mol. Secondary structures determined by NMR for 5' capped 1G and 3G forms of the HIV-1_MAL_ 5' leader (9). For the HIV-1_NL4-3_ strain, secondary structures of DIS, AUG, and U5:AUG were based on NMR studies of the dimeric, noncapped 2G 5'-leader (18, 42). The U5:DIS secondary structure of the HIV-1_NL4-3_ 5' leader was based on NMR studies of a monomeric, noncapped 2G mutant, which identified the [U106-C110]:[G257-G261] helix (18), combined with pairwise sequence alignment of the remaining residues with the HIV-1_MAL_ structure. ΔΔG values represent the differences between the calculated free energies of the idealized and wild-type RNA elements.

^†^Mean ± SD of values for representative HIV-1 strains shown in *SI Appendix*, Fig. S3.

The calculated free energies across all hairpins identified fall into a narrow range (Mean = −17.0 kcal/mol, SD = 1.6 kcal/mol), with a free energy of −17.5 kcal/mol for 57.9% of depositions (Fig. 1*C*). When only considering the 186 unique sequences, the range remains relatively narrow (Mean = −15.4, SD = 3.2 kcal/mol). The consensus structure generated from all analyzed sequences, or from only unique sequences, exhibited noncanonical bulges throughout the 5′ polyA stem (Fig. 1 *D* and *E*, respectively). The presence of bulges is a conserved feature across even phylogenetically distant strains (*SI Appendix*, Fig. S3). To assess the conservation of these structural features we developed a modified Needleman–Wunsch algorithm (43) that aligns base pairs as opposed to nucleotides (*SI Appendix*, Fig. S2 *C*–*D*). Using this algorithm, we aligned either all deposited 5′ polyA hairpin secondary structures or only unique structures with their respective consensus secondary structure and quantified the conservation at each position relative to the consensus structure (Fig. 1 *D*–*E*). Two bulges at positions 12 (uracil) and 17 (cytosine/adenine) downstream of the polyadenylation signal (5′-AAUAAA-3′) were well conserved. The high level of conservation in secondary structure and predicted stability are consistent with proposals for a functional requirement (27, 39).

### Stabilizing the 5′ polyA Hairpin in 3G RNAs Promotes Formation of 1G-like Dimer Structures.

Experiments that probe the function of the 5′ polyA bulges focused on two HIV-1 strains: HIV-1_MAL_ and HIV-1_NL4-3_. Both exhibit transcription start site heterogeneity (2, 9) and have been the focus of structural studies (9, 10, 44) (*SI Appendix*, Fig. S4). Additionally, these strains represent the two predominant DIS palindrome variants (45): 5′-GUGCAC-3′ (found in subtypes A, C, and F, which includes HIV-1_MAL_), and 5′-GCGCGC-3′ (subtypes B and D, which includes HIV-1_NL4-3_) (44, 46). These variants exhibit differences in dimer lability during nondenaturing gel electrophoresis (47). Leaders containing the HIV-1_NL4-3_ palindrome form stable dimers in buffers that lack Mg^2+^, whereas those containing the HIV-1_MAL_ palindrome are kinetically labile and dissociate in the absence of Mg^2+^ (9, 12, 47–49).

**Fig. 2.**
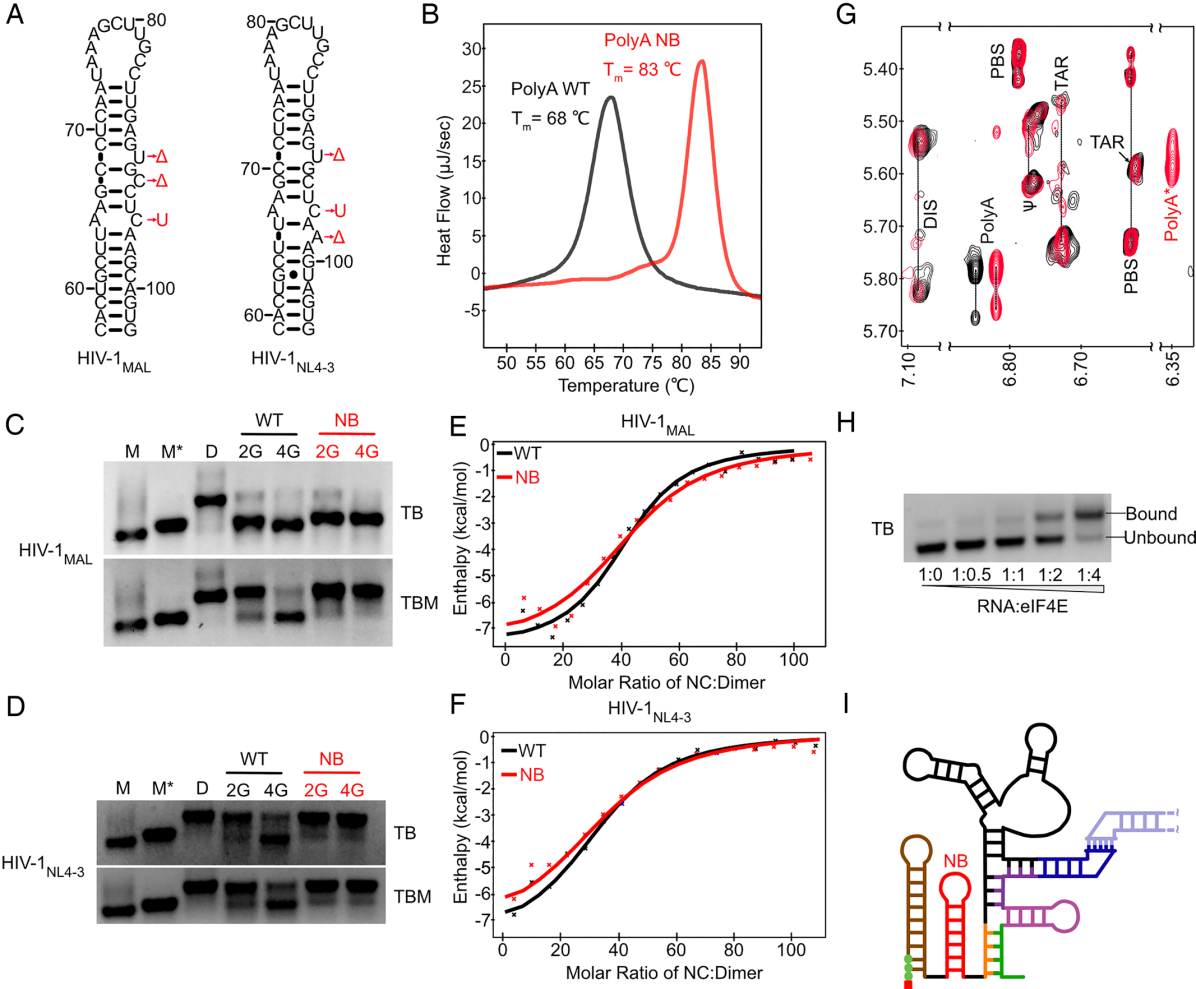
polyA^NB^-modified 3G RNAs form cap-exposed dimers. (*A*) NMR-derived 5′ polyA secondary structures [HIV-1_MAL_ (9) and HIV-1_NL4-3_ (12)] and polyA-stabilizing mutations (polyA^NB^; Δ = deletion, U = uracil substitution. (*B*) Increased melting temperature of the HIV-1_MAL_ polyA^NB^ hairpin (+15 °C; measured by DSC). (*C* and *D*) In vitro dimerization assays for wild-type (WT) and polyA^NB^-mutated (NB) HIV-1_MAL_ and HIV-1_NL4-3_ leader RNAs performed with (TBM) and without (TB) 0.2 mM MgCl_2_ in the gel and running buffer. Controls showing the monomer (M), monomer in dimeric conformation (M*) (9), and dimer (*D*) bands. (*E* and *F*) HIV-1_MAL_ (*E*) and HIV_NL4-3_ (*F*) leader RNAs exhibit similar NC binding properties by ITC. (*G*) Regions of NOESY spectra obtained for HIV-1_MAL_-2G (black) and HIV-1_MAL_ 4G polyA^NB^ (red) leader RNAs prepared with nucleotide-specific ^2^H-labeling (A^2^G^r^U^r^; protons only at adenosine C-2 and ribose and guanosine/uridine ribose positions). Assignments are from ref. 9. polyA* denotes shifted signal due to polyA^NB^ mutations. (*H* and *I*) Dimeric HIV-1_MAL_^Cap^3G polyA^NB^ binds eIF4E (*H*), consistent with an exposed 5′ cap (*I*).

HIV-1_MAL_ and HIV-1_NL4-3_ 5′ leader mutants were generated in which conserved bulges were stabilized or removed (No Bulge, polyA^NB^; Fig. 2*A*). We also determined the melting temperatures (T_m_) of the isolated HIV-1_MAL_ 5′ polyA hairpins by differential scanning calorimetry (DSC). The polyA^NB^ hairpin exhibited a 15 °C increase in T_m_ compared to the native oligonucleotide (Fig. 2*B* and *SI Appendix*, Table S1), consistent with free energy predictions (Table 1). We next studied the dimerization behavior of uncapped RNAs containing an additional 5′-guanosine residue that mimics the 5′ cap of the HIV-1 5′ leader (i.e., 2G RNAs behave similarly to 5′-capped 1G RNAs and 4G RNAs behave similarly to 5′-capped 3G RNAs) (2, 9). As expected, the 2G leader, in which 5′ polyA forms a hairpin in the native sequence, formed a dimer either in the absence or presence of polyA^NB^ stabilizing mutations (Fig. 2 *C*–*D*). However, while the 4G leader containing the native 5′ polyA sequence favored a monomeric conformation, incorporation of polyA^NB^ stabilizing mutations led to dimerization of both HIV-1^MAL^ and HIV-1^NL4-3^ (Fig. 2 *C*–*D*). We used isothermal titration calorimetry (ITC) to test the ability of these dimers to bind HIV-1 nucleocapsid protein (NC), which is responsible for recognition of HIV-1 genomes for packaging (33). We observed NC binding affinities and stoichiometries similar to those of native 2G dimers (Fig. 2 *E*–*F* and *SI Appendix*, Table S2 and Dataset S1). Importantly, nuclear Overhauser effect (NOESY) spectra of the 4G leader with a stabilized 5′ polyA exhibited TAR, PolyA, PBS, DIS, and Ñ± signals that matched those of the 2G dimer containing the native 5′ polyA element (Fig. 2*G*), along with a new upfield shifted signal (polyA*) from the 5′ polyA hairpin due to the stabilizing mutations (Fig. 2*G*). Taken together, our results indicate that 5′ polyA stabilization promotes formation of a dimeric structure with an intact 5′ polyA hairpin, even for a 4G leader predicted to be representative of the native, cap-exposed 3G leader.

To investigate the relative importance of different conserved 5′ polyA bulges, we next generated MAL-4G leader constructs where individual bulges were deleted or modified to promote stabilization (ΔU90, ΔC92, or C95U) (*SI Appendix*, Fig. S5*A*). These modifications increased hairpin melting temperatures by ~5 °C as measured by DSC (*SI Appendix*, Table S1), and the corresponding 4G leader mutant conformers preferentially formed dimers (*SI Appendix*, Fig. S5*B*).

We considered the possibility that these effects may be mediated by monomer destabilization rather than dimer stabilization. Previous structural models have predicted that residues U90, C92, and C95 may play important roles within the extended U5-PolyA:DIS helix (*SI Appendix*, Fig. S5*C*) (9), suggesting that our mutations that promote 4G leader dimerization may do so by interrupting structures that sequester the DIS. Therefore, we generated a complementary set of constructs designed to stabilize the 5′ polyA hairpin while avoiding interference with the monomer structure. We compared nucleotide insertions +A69 and +G68, which are inserted into a region that is unstructured in the monomer and are complementary to the U90 and C92 bulges respectively, to ΔU90 and ΔC92 leaders (*SI Appendix*, Fig. S5*A*). We also introduced a rescue mutation (G288A) to the C95U leader, designed to restore expected base pairing in the monomer (*SI Appendix*, Fig. S5*C*). These mutations all promoted dimerization for the 4G leader, suggesting that monomer destabilization is not a major factor in driving the shifted structural equilibrium (*SI Appendix*, Fig. S5*D*).

Interestingly, the +A69 mutant was less competent for dimerization than other mutants despite contributing similar stabilizing effects (~3 kcal/mol), raising the possibility that monomer destabilization may play some role in promoting dimerization in the ΔU90 leader. Another possibility is that the position of the bulges and mismatches is important, with residues closer to the hairpin loop influencing dimer stabilization less than those in the stem. To test these possibilities, we generated a leader with an additional C-G base pair at the top of the stem which also induces a ~3 kcal/mol stabilizing change. This 3G leader mutant (which behaves similarly to a 4G leader) remained monomeric (*SI Appendix*, Fig. S5*E*). Our results thus support a model wherein HIV-1 evolved 5′ polyA bulges of appropriate number and in the proper location needed to control hairpin stability and enable the structural plasticity needed to allow viral RNA dimerization and Gag binding.

### The 5′ Cap is Exposed in Dimeric 3G Mutants with Stabilized 5′ polyA Hairpins.

In native 1G transcripts, the 5′ polyA hairpin stacks coaxially with the TAR hairpin, thereby sequestering the 5′ cap (3, 8, 9). To determine the influence of 5′ polyA hairpin stabilization on cap exposure in 3G RNAs, we tested 5′-capped (50) 3G HIV-1_MAL_ leader RNAs with polyA^NB^ mutations (3G-NB) for their ability to bind the cellular cap-binding protein eIF4E (9). Electrophoretic mobility shift assays were conducted in the absence of magnesium in the gel and running buffer as previously described (3, 9). Under these conditions, HIV-1_MAL_ dimers dissociated into monomers that retain the conformation found in dimeric RNA (Fig. 2*C*) (9). Remarkably, the 5′-capped 3G-NB leader, which adopts a 1G-like dimer structure, exhibited eIF4E binding properties similar to those of the native (and monomeric) 5′-capped 3G RNA (Fig. 2*H*). The 5′-capped 1G RNAs do not bind eIF4E under similar conditions (9). Taken together, our results reveal that 5′ polyA stabilization induces formation of a dimeric leader structure independent of transcription start site regulation, and that cap exposure in these constructs is independent of 5′ polyA structure (Fig. 2*I*). The 5′-capped 3G-NB leader therefore decouples cap-sequestration from 5′ polyA structure and monomer:dimer equilibrium, allowing us to evaluate the relative roles of dimerization and cap exposure on biological functions including selective RNA packaging and translation.

### Cap Exposure Regulates RNA Packaging Irrespective of 5′ polyA Element Conformation.

To investigate the relative importance of cap sequestration, 5′ polyA stability, and monomer:dimer equilibrium to HIV-1 genome packaging, we compared the packaging efficiencies of RNAs containing cap-exposed 3G and 3G-NB leaders in competition with those containing cap sequestered 1G and 1G-NB leaders, respectively. Cultured 293 T cells were transiently transfected with HIV-1 vectors containing the native HIV-1_NL4-3_ U3 sequence followed by the first 368 nucleotides of HIV-1_MAL_ or HIV-1_NL4-3_ followed by the HIV-1_NL4-3_ *gag/pol* sequence. Vectors either contained the native 5′ polyA hairpin sequence or the NB mutations to stabilize the 5′ polyA hairpin (Fig. 2*A*). Selective packaging was monitored by comparing the 5′ end sequences of cellular and virion-associated RNA transcripts as determined by cap-dependent adaptor ligation assays (51). Transcription start sites were determined by comparison to 2G and 4G RNA controls.

**Fig. 3.**
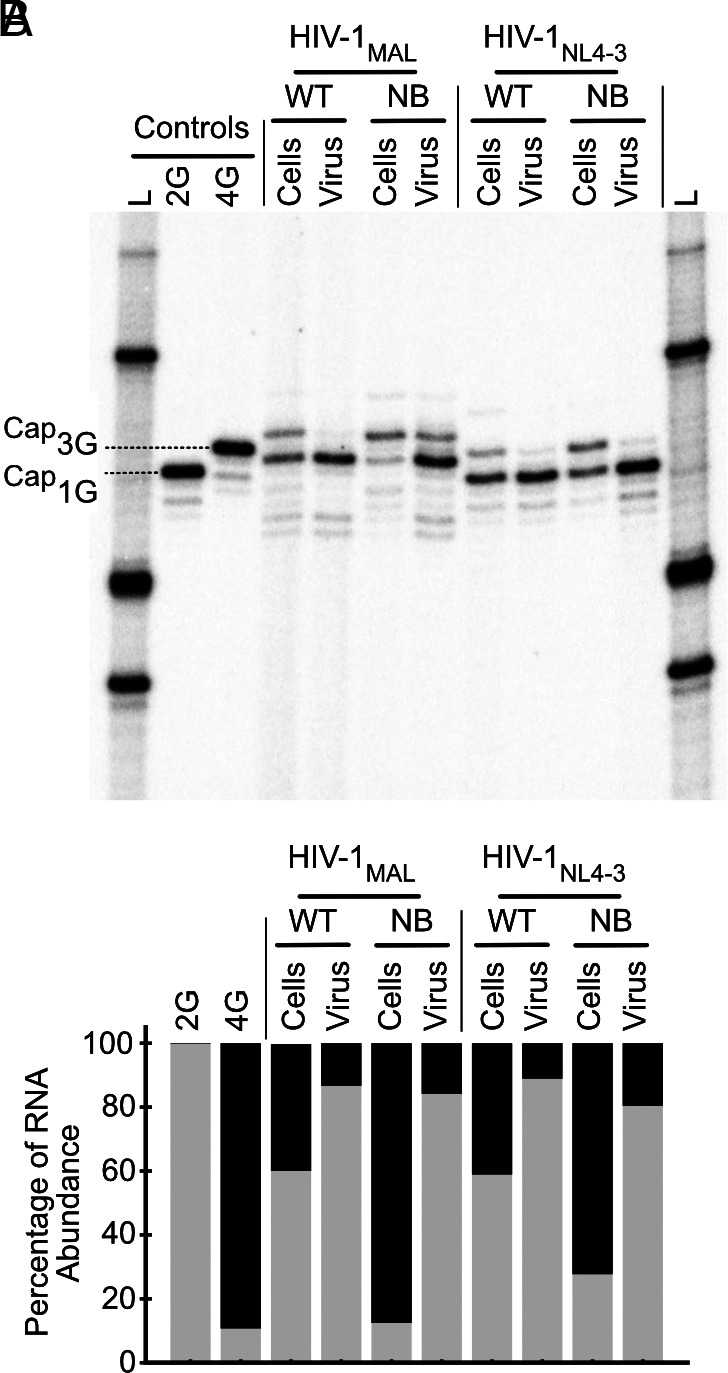
Dimeric, cap-exposed 3G polyA^NB^ RNAs are not competitively packaged. (*A*) In vitro packaging assay via cap-dependent adaptor ligation assay comparing RNA proportions in cells to that in virus. Single nucleotide resolution allows discrimination of RNA start via comparisons to controls corresponding to HIV-1_NL4-3_ 5′-capped 1G (2G) and HIV-1_NL4-3_ 5′-capped 3G (4G). Note that a 5′ cap leads to incorporation of a G in the final PCR product obtained using the cap-dependent adaptor ligation assay employed; thus, 5′ capped 1G RNA and 3G RNAs generate products with two and four 5′ guanosines, respectively, which exhibit mobilities of noncapped 2G and 4G controls (*Left*-most lanes). (*B*) Densitometric quantification of packaging propensities (1G and 3G RNAs shown in gray and black, respectively).

In all experiments, 1G and 3G RNA transcripts had detectable expression in the cell lysate (Fig. 3 *A* and *B*). However, the relative amount of cellular 1G and 3G RNA detected varied, with 3G transcripts in higher abundance for leaders containing a stabilized 5′ polyA hairpin. This may be due to a previously unrecognized role that the 5′ LTR DNA sequence has on transcription start site selection or possibly an unknown influence of dimerization on translation (51, 52). In each case, we assessed packaging efficiency by comparing RNA levels in cells versus virions. For leaders containing the native 5′ polyA hairpin sequence, 1G transcripts were enriched within virions for both HIV-1_MAL_ and HIV-1_NL4-3_ (Fig. 3 *A* and *B*), in agreement with previous observations (2, 9). This preference for packaging 1G leaders was maintained when the 5′ polyA hairpin was stabilized in both HIV-1^MAL^ and HIV-1^NL4-3^, even though the 5′-capped 3G polyA^NB^ mutants exhibited dimerization and NC binding properties similar to those of the capped 1G constructs (Fig. 2 and 3 *A* and *B*). These findings confirm that dimerization, 5′ polyA stability, and NC binding alone are insufficient to drive efficient HIV-1 genome packaging and highlight the importance of cap sequestration for competitive packaging.

### Cap Exposure Promotes Efficient HIV-1 RNA Translation Regardless of 5′ polyA Status.

**Fig. 4.**
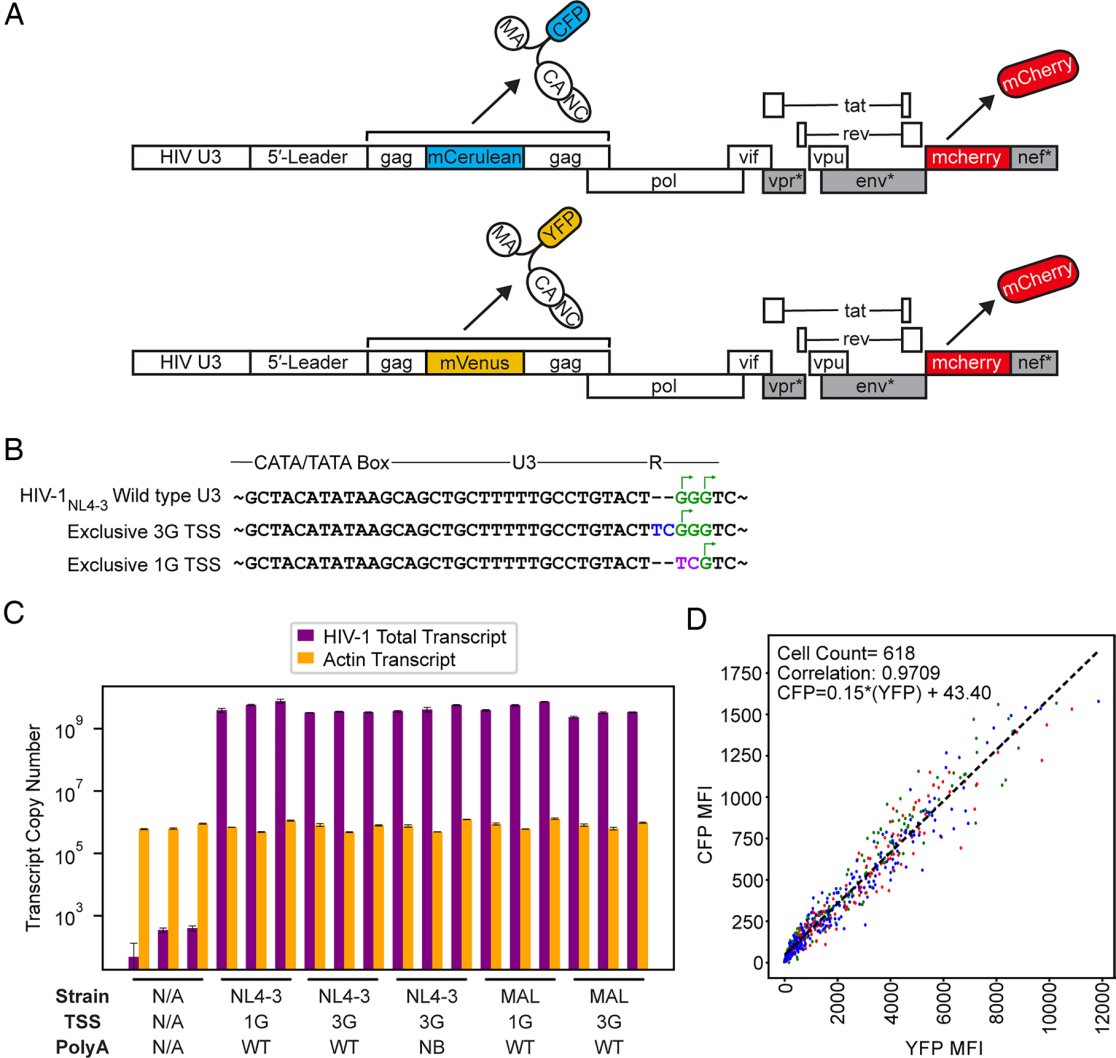
In-cell competitive assay for evaluating the effect of TTSS on translation. (*A*) Three color reporter system for monitoring competitive translation via cotransfection of two separate full-length proviral plasmids. Leader-driven translation was monitored by measuring changes to Gag-CFP or Gag-YFP expression over time, with mCherry also expressed by each virus as a fluid phase marker for cell tracking (* denotes inactivating mutations within gene regions intended to prohibit viral replication in vivo, for biosafety). (*B*) Mutations in U3 that control TSS usage (51) showing residue additions (blue), substitutions (purple), and potential and actual transcription start sites (green and arrows, respectively). (*C*) RT-qPCR assays for quantification of HIV-1 total transcript production (55). Error bars denote SD from three separate experiments. (*D*) Example of YFP versus CFP mean fluorescence intensities (MFI) used to calculate correlation coefficients (NL43-3G-YFP versus NL43-3G-CFP control at 30 h posttransfection). Each point represents an individual cell identified by Cellpose (56) with colors based on the three regions of interest (ROI) collected for each well. The best fit linear trendline is shown.

In cells, spliced HIV-1 transcripts were recently shown to be enriched in 3G species relative to 1G RNAs (53), which is consistent with the known general role of the 5′ cap in promoting splicing (54). To investigate the role of 5′ leader structure on translation, we developed a three-color live cell imaging strategy in which viral Gag protein expression is monitored in cells based on single cell measures of fluorescently tagged versions of Gag fused to either cyan fluorescent protein (Gag-CFP, Gag fused to mCerulean) or yellow fluorescent protein (Gag-YFP, Gag fused to mVenus), with both constructs expressing mCherry from the viral *nef* locus as a fluid-phase marker used for cell tracking (Fig. 4*A*). In this assay, Gag-CFP and Gag-YFP viruses bearing modified leader regions were coexpressed to monitor competitive translation between RNA transcripts based on comparative levels of YFP and CFP fluorescence in single cells over time (*SI Appendix*, Fig. S6*A*).

**Fig. 5.**
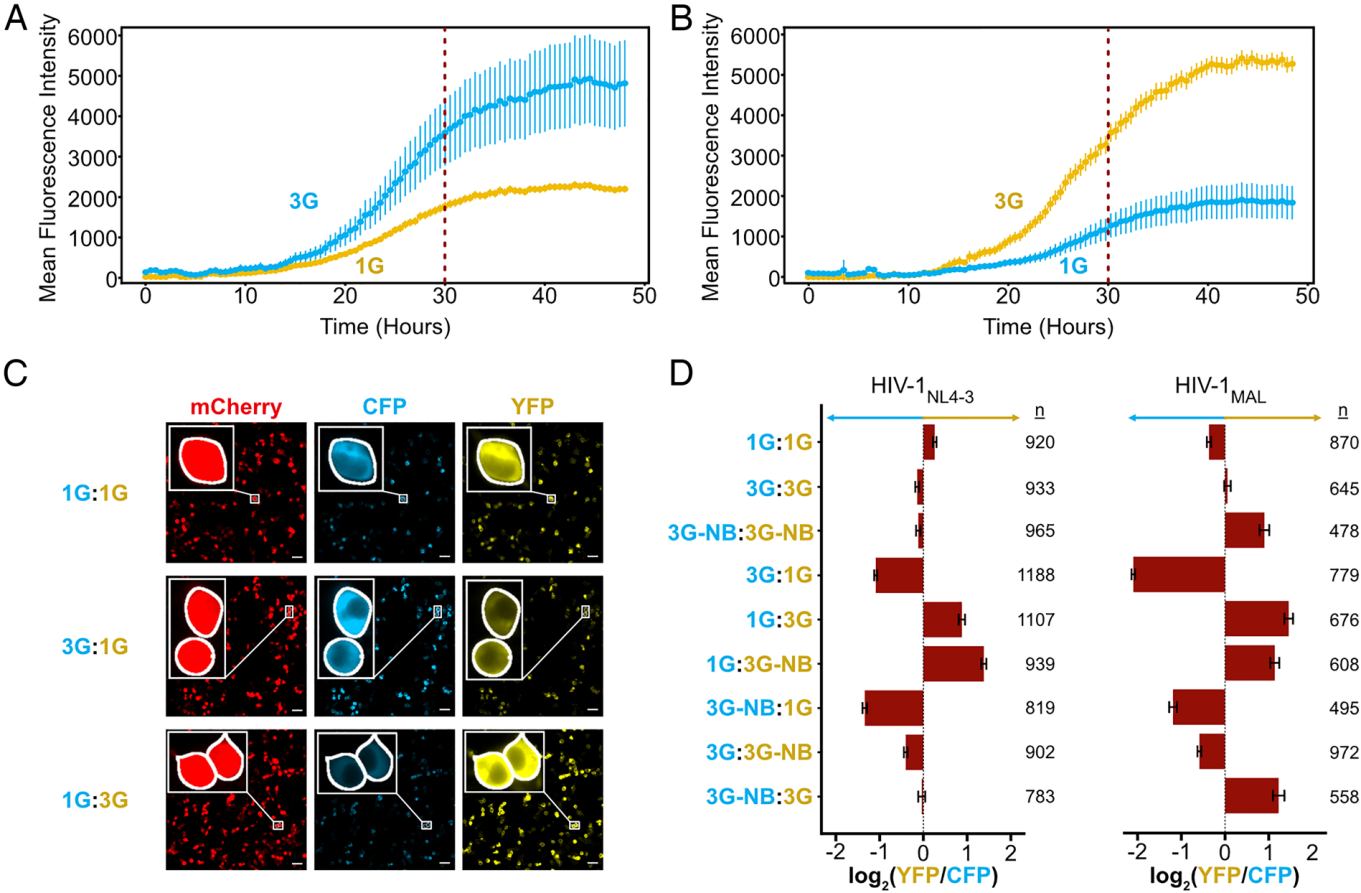
Cap-exposed RNAs exhibit enhanced translation efficiencies. (*A* and *B*) Average mean fluorescent intensity across all cells identified at each timepoint in CFP (blue) and YFP (yellow) channels for NL43-3G-CFP in competition with NL43-1G-YFP (3G:1G) (*A*) and an inverted experiment showing NL43-1G-CFP in competition with NL43-3G-YFP (1G:3G) (*B*). Error bars represent the SEM of the MFI at each time point. 30-h time point used for subsequent analysis indicated by a dashed line. (*C*) Representative images at 30 h post transfection for a control (NL43-1G-CFP vs NL43-1G-YFP, 1G:1G), 3G:1G, and 1G:3G in mCherry, CFP, and YFP channels. Insets show zoomed (10×) images of individual cells identified by Cellpose, which highlights similar CFP and YFP detection in our control and differences between channels in 1G:3G and 3G:1G images. White scale bars represent 50 μm. (*D*) Average log_2_(YFP/CFP) ratios for single cells 30 h post transfection across two separate transfections normalized for differences in YFP and CFP using control samples from matching transfections (Supplemental Information). Error bars show the 95% CI of the mean across all cells from two separate transfections (n = number of cells).

The core promoter elements that drive transcription start site heterogeneity have recently been identified, together with mutations within the U3 promoter region that exclusively produce 1G or 3G transcripts (Fig. 4*B*) (51, 52). We incorporated these mutations within our three-color reporter system alongside either native or polyA^NB^ sequences. These mutations did not alter HIV-1 RNA transcription levels as detected by RT-qPCR (Fig. 4*C*). Four hours following cotransfection, YFP, CFP, and mCherry fluorescence intensity was monitored on a per-cell basis every 30 min for 48 h as described in Methods and Supplementary Methods (Fig. 4*D*). Using the U3 promoter mutations to control transcription start site in cells, we were able to compare translation propensities of 3G and 1G leaders in both HIV-1_MAL_ and HIV-1_NL4-3_. 3G RNAs, which form monomers with an exposed cap moiety, were translated at significantly higher levels than 1G RNAs, which form dimers and sequester the cap (Fig. 5 *A*–*C* and Movies S1 and S2). This preference was independent of fluorophore (Fig. 5 *A*–*C*) and seen for both HIV-1_MAL_ and HIV-1_NL4-3_ leader sequences (Fig. 5*D*). This observation supports the hypothesis that transcription start site regulates mRNA translation (51, 52), likely due to 3G transcripts more efficiently recruiting cap-dependent initiation factors (57).

To further investigate the structural basis for this preference, we compared translation of 3G-NB RNAs with 1G RNAs, which have the same secondary structure and dimerization properties but with an exposed rather than sequestered cap. Despite their similar structures, 3G-NB RNAs outcompeted 1G RNAs (Fig. 5*D*). These results were consistent with cap exposure serving as a positive regulator of translation while simultaneously acting as a major negative regulator of packaging, with these effects governed by 5′ polyA stem-loop stability and operating independently of other leader RNA structures that govern genome dimerization and Gag binding. We note that in all experiments, 1G RNA translation levels were reduced but not fully abrogated, suggesting that 5′ cap-sequestered RNAs are translation-competent, but at lower efficiencies relative to cap-exposed RNAs. We hypothesize that coaxial stacking of the TAR-polyA hairpin is dynamic, with transient cap-exposed intermediates allowing for irreversible capture by the RNA translation machinery.

To investigate whether 5′ polyA stability regulates translation efficiency in cap-exposed RNAs, we compared translation of 3G (monomeric) and 3G-NB (dimeric) RNAs, each of which has an exposed cap, but which differ in their 5′ polyA stability and dimerization properties. For RNAs containing HIV-1_NL4-3_ derived leaders, a small preference was seen for 3G monomers, but the effect was significantly smaller than when comparing cap-exposed and cap-sequestered RNAs (Fig. 5*D*). Similar trends were observed for HIV-1_MAL_ RNAs, suggesting that RNA structure and 5′ polyA stability are secondary to cap exposure in determining translation efficiency.

### Other Elements in the HIV-1 5′ Leader Evolved Similar Free Energy Depressions.

5′ polyA is one of several RNA elements that adopt different secondary structures depending on the presence of either one or three 5′ guanosines. As shown by NMR for the HIV-1_MAL_ 5′ leader, U5 base pairs with AUG and DIS forms a hairpin structure in the dimeric 1G 5′ leader, whereas U5 base pairs with DIS and AUG forms a hairpin in the monomeric 3G 5′ leader (9) (Fig. 1). Similar structures are likely formed by the 5′-capped 3G and 1G forms of the HIV-1_NL4-3_ 5′-leader, based on NMR studies of monomeric and dimeric forms of uncapped 2G RNAs (9, 18, 58). We compared predicted secondary structures and free energies of the DIS, AUG, U5:AUG, and U5-DIS elements across the same evolutionarily divergent HIV-1 strains shown in *SI Appendix*, Fig. S3 for 5′ polyA. For each domain, we utilized the boundaries established by previous NMR studies of the MAL leader monomer (AUG and PolyA-U5:DIS) and dimer (DIS and U5:AUG) (summarized in Dataset S2). For comparison, we determined free energies of idealized RNA structures that lacked noncanonical base pairs, bulges, and G:U wobbles for both MAL and NL4-3 strains, Table 1. Each of the MAL and NL4-3 wildtype sequences exhibited free energies that are significantly depressed (ΔΔG = 8.4 to 21.3 kcal/mol) relative to that of the corresponding idealized structure. Additionally, average free energy values of each element showed small SD (0.5 to 4.2 kcal/mol) across evolutionarily distant viruses, highlighting the conservation of thermodynamic parameters across all strains examined (Table 1 and *SI Appendix*, Table S3).

## Discussion

HIV-1 genomes are selected for packaging as dimers, and there is considerable evidence that dimerization-dependent RNA structural differences modulate gRNA versus mRNA functions (13, 15, 17, 38, 59–61). Dimerization appears to be controlled by a TTSS mechanism (2), in which the 1G leader adopts a dimeric structure that exposes ~20 high-affinity NC binding sites (33) while simultaneously sequestering the 5′ cap (3) and residues important for splicing and translation (gRNA) (11). In contrast, the 3G leader adopts a remodeled monomeric structure that sequesters residues that promote RNA dimerization while exposing the cap and residues important for viral mRNA splicing and translation (mRNA) (9). Because the cap is base-paired in the 1G leader but not in the 3G leader, the 3G leader contains a single additional G-C base pair compared to the 1G leader. How does such an energetically modest modification (~3 kcal/mol) promote global remodeling of the ~350 nucleotide 5′-leader?

The present studies focused on the 5′ polyA element of the 5′ leader because the additional terminal guanosine of the 3G transcript interacts exclusively with this element and because early studies showed that mutations that stabilize or destabilize the hairpin beyond its wild-type stability dramatically reduced viral replication (27, 28, 34). We confirmed by a structure-based phylogenetic analysis that HIV-1 evolved 5′ polyA hairpins with a narrow window of free energies (−17.0 ± 1.6 kcal/mol for all 1,268 depositions; −15.4 ± 3.2 kcal/mol for 186 unique sequences) that are well above the free energy of consensus hairpins lacking the bulges (Fig. 1*C* and Table 1). The destabilizing bulges are conserved across evolutionarily distant strains of HIV-1, supporting the hypothesis that they are required for tuning the stability of the 5′ polyA hairpin (35, 36). Importantly, 3G leader RNAs containing the polyA^NB^ mutations exhibited dimerization propensities, NMR spectral features, and NC binding properties similar to those of the native 1G leader (Fig. 2), supporting our hypothesis that changes in 5′ polyA structure, induced by a single G-C base pair, serves as the trigger for both local and global structural changes (2, 9). Thus, the ability of the 5′ polyA element to adopt an extended structure in 3G transcripts is critical for preventing RNA dimerization and associated structural changes that promote Gag binding and cap sequestration.

Although the 1G and 3G-NB leader RNAs exhibit similar dimerization and NC-binding propensities, they differ in their ability to bind the cellular cap-binding protein eIF4E (Table 2). Native 1G RNAs do not bind eIF4E due to structural sequestration of the 5′ cap, which is sandwiched between coaxially stacked TAR and 5′ polyA helices (9). In contrast, the native and polyA^NB^-modified 3G leaders readily bind eIF4E. NMR studies have shown that the 5′ cap is exposed and conformationally labile in the native 3G leader, and it is likely that the additional guanosines in the 3G-polyA^NB^ leader promote cap exposure either by preventing end-to-end stacking of the TAR and polyA^NB^ hairpins or by simply extending the cap away from the stacked hairpins. Regardless, stabilization of the 5′ polyA hairpin in both MAL and NL4-3 derived 3G transcripts promoted both dimerization and exposure of Gag binding sites but did not promote packaging to levels observed for the 1G transcript with similar dimerization and NC binding properties (Figs. 2 and 3). Similar results were observed for a construct containing an extended 5′ terminus [Cap-G(-2)-A(-1)-A(1)-G(2)-G(3)-], which also forms dimers and exposes Gag binding sites but is poorly packaged due to exposure of the 5′ cap (3). These findings collectively indicate that dimerization and Gag binding are insufficient to overcome the dominant negative effect of 5′ cap exposure on genome packaging.

**Table 2.** Influence of transcriptional start site usage and polyA stability on HIV-1_MAL_ 5′ Leader structure and function^*^

	5’ polyA structure	5’ cap exposure	Dimerization	NC Binding	Packaging efficiency	Translation efficiency
**WT-1G**	hairpin	sequestered	dimer	high	high	low
**WT-3G**	extended	exposed	monomer	not determined	low	high
**NB-3G**	hairpin	exposed	dimer	high	low	high

^*^In vitro 5’ polyA structure, 5’ cap exposure, dimerization propensity, and NC binding for wild-type (WT) 1G and 3G RNAs are from ref. 9.

The influence of a different set of 5′ polyA mutations on packaging was recently investigated using 5′-rapid amplification of cDNA ends (RACE) and a packaging assay involving competitive constructs with different DIS sequences (10). Although the RNA structures and proposed packaging mechanisms differ, the packaging data appear consistent with our findings. Mutations designed to stabilize the lower 5′ polyA stem while destabilizing base pairs in the monomeric conformer favored in vitro dimerization and did not affect 1G packaging selectivity, as expected. Mutations designed to destabilize the base of the 5′ polyA hairpin inhibited both dimerization and selective 1G packaging, and addition of nonnative residues between the TAR and 5′ polyA hairpins that would be expected to disrupt stacking and promote cap exposure also abrogated selective 1G packaging. These findings are consistent with our previously proposed bipartite packaging mechanism, wherein efficient packaging requires both RNA dimerization to expose Gag binding sites, and sequestration of the 5′ cap to prevent capture by the cellular RNA processing and translation machinery (3).

Whereas the requirement of RNA dimerization for packaging is well known, its potential influence on RNA processing or translation is less understood. In vitro studies suggested that dimerization attenuates splicing (61) but does not affect translation (28, 38, 62). However, these studies were conducted with transcripts that did not account for 5′-end heterogeneity. More recently, mutations that destabilize 5′ polyA were shown to increase mRNA translation in an in-cell assay conducted with a normal HIV-1 promoter that produces both 1G and 3G transcripts (62). Here, we were able to monitor relative translation efficiencies of 1G versus 3G RNA species in cells cotransfected with constructs that encode differentially labeled products of the 1G and 3G transcripts. We found that native 3G transcripts are translated with greater efficiency than native 1G transcripts (Table 2), which correlates with the preference of 3G RNAs to adopt structures with an exposed cap. All mutant constructs that favored 5′ cap exposure, including those with 5′ polyA mutations that also promote dimerization and NC binding, were translated more efficiently than those favoring structures that sequester the 5′ cap. Similar results were obtained for both HIV-1_MAL_ and HIV-1_NL4-3_ derived constructs. Thus, in contrast to packaging, 5′ cap exposure is a dominant positive determinant of translation. Except for its influence on 5′ cap exposure, dimerization does not appear to significantly influence translation efficiency.

In summary, we have shown that transcripts with an exposed 5′ cap are both more efficiently translated and less efficiently packaged than those with a sequestered 5′ cap, regardless of the propensity of the RNA to form dimers or bind NC (Table 2). Our findings support a mechanism in which TTSS-mediated dimerization modulates both packaging and translation functions: native 1G transcripts form dimeric structures that expose high-affinity NC binding sites and concomitantly sequester the 5′ cap, both of which are required for efficient RNA packaging. In contrast, 3G transcripts adopt monomeric structures with exposed 5′ caps that promote capture by eIF4E and the cellular translation machinery. Structural plasticity is dependent on evolutionarily conserved bulges and noncanonical base pairs in the 5′ polyA element that reduce its stability relative to idealized hairpins. Noncanonical base pairs and bulges are also present in other conserved secondary structures formed by the 1G and 3G 5′-leader RNAs, resulting in formation of helices with depressed stabilities relative to idealized helices (Table 1). Notably, mutations that stabilize the U5:AUG helix and promote dimerization also promote competitive RNA packaging, whereas mutations that destabilize U5:AUG inhibit both dimerization and packaging (18). This behavior parallels that observed here, where the mutations that stabilize the polyA hairpin promote dimerization and packaging. In fact, residues throughout the 5′ leader have been shown by mutational interference to influence dimerization (37). Thus, we do not believe that 5′ polyA stability guides genome packaging (10), but instead that elements throughout the 5' leader have evolved stabilities tuned to enable a single G-C base pair to control global RNA structure and function.

## Materials and Methods

### Structural Conservation Analysis.

All HIV-1 complete genome sequences (20,439 depositions) present within the Los Alamos National Lab HIV Compendium (40) as of March 2, 2024, were extracted. Annotation of the *gag* start codon allowed identification of the 5′-LTR, which was missing in many of the depositions. A total of 1,268 5′ polyA hairpins were identified within the 5′-LTR using an algorithm relying on the presence of the polyadenylation signal and on free energy minimization to better identify the boundaries of the hairpin as described in Supplemental Methods. A phylogeny tree of the representative 5′ polyA hairpin sequence for each subtype was generated using an alignment of the envelope gene (63, 64) using CLUSTALW (65) with bootstrapping (n = 1000) using PhyML (66). Free energies of the identified hairpins reported were determined by the RNAfold program in the ViennaRNA software suite (67). A consensus secondary structure from the 186 unique 5′ polyA hairpin sequences identified was inferred using the locARNA software tool (19–21). Alignment to this consensus structure was based upon the Needleman–Wunsch algorithm (43) that we modified to align structural elements. Further details on this bioinformatic analysis for 5′ polyA and other domains can be found in the “Structural Conservation Analysis” section within Supplemental Information.

### Preparation of RNAs for In Vitro Biophysical Studies.

Details regarding the preparation and purification of uncapped and 5′ capped RNAs for in vitro biophysical studies are described in Supporting Information. All constructs and primers utilized are found in *SI Appendix*, Tables S4–S7.

### Differential Scanning Calorimetry (DSC) of 5′ polyA Hairpin Constructs.

DSC experiments were conducted on isolated 5′ polyA hairpins produced via in vitro transcription as described above. Purified RNAs were dialyzed overnight in 50 mM Potassium Phosphate buffer (pH 6.5) in accordance with DSC sample preparation procedures as described previously (68). Samples were diluted to 50 μM based on UV absorbance, degassed under vacuum for 15 min, and then loaded onto the calorimeter with phosphate buffer (50 mM phosphate, pH 6.5) as the reference solution. DSC experiments were conducted on a Nano DSC instrument (TA Instruments). During DSC experiments samples were pressurized to 3 atm and then heated from 5 to 95 °C at a rate of 1 °C/minute. Data were analyzed using NanoAnalyze software (TA instruments) and fitted with a two-state scaled model to give Aw, T_m_, and ΔH, as described previously (68). Fitted values were then subjected to Monte Carlo simulation with 100 trials to calculate a 95% CI for each fitted parameter. Values and CI for all hairpins tested are found in *SI Appendix*, Table S1.

### Isothermal Titration Calorimetry.

All ITC experiments were performed using a MicroCal PEAQ-ITC Automated isothermal titration calorimeter (Malvern Panalytical). RNA and Nucleocapsid protein (NC) samples were dialyzed overnight in ITC buffer (20 mM MOPS, pH 7.5, 140 mM KCl, 10 mM NaCl, 5 mM MgCl_2_, and 5 mM TCEP). MOPS buffer was used to avoid sequestration of zinc (II) and magnesium (II) ions that can occur in Tris and phosphate buffers (69). Nucleocapsid was prepared in house as described previously (33, 70) and supplemented with 100 μM ZnCl_2_ predialysis. 40 µL of 200 to 250 µM NC was loaded to the injection syringe. The calorimetry cell was loaded with 200 uL of RNA at ~1 μM. Experiments began with thermal equilibration at 25 °C followed by a 60-s initial delay. The titration began with a single injection of 0.4 μL followed by 18 serial injections of 2 μL with 120-s delays between injections. Composite controls were used to correct data where protein-to-buffer and buffer-to-RNA controls were subtracted from data, while buffer-to-buffer controls were added. Data were baseline corrected through manual identification of peaks. MicroCal PEAQ ITC analysis software (Malvern) was used for baseline correction, integration, and curve fitting to extract stoichiometries, affinities, and thermodynamic parameters (*SI Appendix*, Table S2 and Dataset S1).

### Native Gel Electrophoresis (In Vitro Dimerization Assays and eIF4E EMSAs).

RNA samples (0.1 to 10 μM) were prepared in water and heat denatured at 100 °C for 5 to 10 min and then snap cooled on ice to reduce RNA oligomerization. Samples were incubated in either a potassium phosphate-based physiological ion (PI) Buffer (10 mM Potassium phosphate, pH 7.4, 140 mM KCl, 1 mM MgCl_2_) or a Tris-based PI Buffer (20 mM Tris, pH 7.5, 140 mM KCl, 1 mM MgCl_2_) at 37 °C overnight. Protein samples were diluted in PI buffer and incubated at room temperature for 1 h. For gel shift assays eIF4E protein was produced in-house as previously described (3). Protein and RNAs were mixed at the indicated ratios and incubated for 2 h. Samples were diluted with 10× native agarose gel loading solution (0.17% bromophenol blue and 40% (vol/vol) sucrose) or 50% glycerol before loading. Gels were loaded with 50 to 500 ng of RNA on 1 to 2% agarose gels prestained with ethidium bromide. Gels were run at 115 V for 1 to 2 h in 1X TB (44.5 mM Tris-Boric Acid, pH 7.5) or 1X TBM (44.5 mM Tris-Boric Acid, 0.2 mM MgCl_2_, pH 7.5).

### NMR Spectroscopy Data Collection and Analysis.

Samples for NMR studies of leader RNAs (~100 μM) in D_2_O [99.8%; CIL] were prepared in PI buffer (10 mM KH_2_PO_4_, pH 7.4, 1 mM MgCl_2_, 140 mM KCl) and placed in shaped sample tubes (Bruker). Data were collected with a Bruker AVANCE IV spectrometer (800 MHz ^1^H, 35 °C), nonexchangeable ^1^H assignments were obtained from 2D NOESY data (NOE mixing time = 650 ms, relaxation delay = 5 to 10 s, T = 35 °C). All NMR data were processed with NMRFx Analyst and analyzed with NMRViewJ (71, 72). Chemical shift assignments were made with reference to previously published assignments, using NMRViewJ assignment tools (9, 12, 73).

### Packaging Assay.

Plasmid construction, virus production, and RNA content analysis were conducted as previously described (51). Constructs were generated for HIV-1_NL43_ and HIV-1_MAL_ with and without mutations in the 5′ polyA region using overlapping PCR.

### Plasmids for Translation Assays.

The construction of full-length HIV-1 proviral plasmids of the pNL4-3 molecular clone with inactivated *env, vpr,* and *nef* genes and expressing Gag-YFP and mCherry from the *nef* locus was as described previously (74, 75). For the competitive assay, Gag-YFP/mCherry virus was used to generate a second Gag-CFP/mCherry virus plasmid. Mutations to control the RNA transcription start site, 5′ polyA structure, and 5′ leader strain were incorporated by overlapping PCR using Phusion High Fidelity DNA Polymerase (New England Biolabs) with appropriate Puc57 plasmids from *SI Appendix*, Table S4 for insertion into proviral plasmids. Primers used are detailed in *SI Appendix*, Table S8. Mutagenesis reactions were transformed into Stbl2 MAX efficiency competent cells (Invitrogen) and screened for insertion using colony PCR. Successful mutagenized plasmids were identified via whole plasmid sequencing (Eurofins) and were subsequently purified at midiprep scale with endotoxin removal (ZymoPURE II Maxiprep Kit).

### Cell Culture for Translation Assays.

Human embryonic kidney (HEK) 293 T cells were obtained from the American Type Culture Collection (Manassas, VA). Cells were grown at 37 °C, 50% humidity, and 5% CO_2_ in Dulbecco’s modified Eagle medium (DMEM) supplemented with 10% heat-inactivated fetal bovine serum (FBS) and 1% penicillin-streptomycin-L-glutamine. 6,000 cells were plated in each well within 18-well ibidi µ-slides and cotransfected using polyethyleneimine in Opti-MEM (3 μL PEI: 100 μL Opti-MEM: 1 μg DNA) with 52.5 ng of each plasmid for 4 h before beginning data collection. Transfection media remained on cells throughout data collection to maximize protein expression.

### Microscopy and Image Analysis for Translation Assays.

Imaging was performed using a Nikon Ti-Eclipse inverted wide-field microscope (Nikon Corp, Minato, Tokyo, Japan) and a 20x Plan Apo objective lens (NA, 0.75). Image acquisition was performed using an Orca-Flash4.0 digital complementary metal oxide semiconductor (CMOS) camera (Hamamatsu Photonics, Skokie, IL) and Nikon NIS Elements software (ver. 4.30.02). Differential interference contrast (DIC) images were collected together with the following fluorescence excitation/emission filter sets: 325 to 375/435 to 485 nm, 490 to 510/520 to 550 nm, and 565 to 590/590 to 650 nm. Three fields of view were acquired for each condition with 50% light intensity. Images were processed and analyzed using the Fiji/ImageJ2 software (76) with the assistance of BaSiC (77) and Cellpose (56) plugins.

For our cell-based translation assays, the raw data were filtered to ensure cotransfection of both plasmids encoding the Gag-CFP and Gag-YFP viruses (Fig. 4*D* and *SI Appendix*, S6 *B*–*D*) and corrected for YFP and CFP detection sensitivity (*SI Appendix*, Fig. S7, Movie S3) as described in Supplemental Information. Real-time imaging showed that increases in average cellular YFP and CFP fluorescence over time exhibited near linear growth between 12 and 36 h (Fig. 5 *A* and *B*), so we chose 30 h as a representative timepoint for our comparative analyses (Fig. 5 *A*–*C*) (ratios are shown to be relatively consistent across timepoints *SI Appendix*, Fig. S8). To this end, log_2_(YFP/CFP) ratios were calculated for all transfected cells and averaged for each condition at 30 h to determine which leader outcompeted in translation, testing each 5′ leader variant in both the Gag-CFP and Gag-YFP background. Further details of image analysis can be found in “Live Cell Imaging Analysis” in supplemental information. All data and statistical analyses can be found in Datasets S3 and S4.

### RNA Isolation and RT-qPCR Analysis.

Cell culture and transfection were performed as described for translation assays, except cells were cotransfected with 52.5 ng of appropriate full-length proviral mVenus plasmid and 52.5 ng of pCMV-3xHA-CFP plasmid. After 48 h cells were washed and resuspended in 1× Phosphate-Buffered Saline (PBS), and then pelleted at 300×g for 5 min. RNA was extracted using the Direct-Zol RNA miniprep kit (Zymo). RT-qPCR for total HIV-1 transcript was done as previously described using the iTaq Universal One-Step RT-qPCR kit (Bio-Rad) and primers listed in *SI Appendix*, Table S9 (55). Actin primers and standard curve templates were derived from pDsRed-Monomer-Action PT3827-5 (Clontech Laboratories, Inc). Measurements were conducted via Bio-Rad CFX Duet Real-Time PCR System.

## Supplementary Material

Appendix 01 (PDF)

Dataset S01 (CSV)

Dataset S02 (CSV)

Dataset S03 (CSV)

Dataset S04 (CSV)

Movie S1. Representative movie of NL43-3G-CFP in competition with NL43-1GYFP. Images in the mCherry, YFP, and CFP channels were collected every 30 minutes for 48-hours. Movies include 7 frames per second. CFP channel was normalized to account for detection differences.

Movie S2. Representative movie of NL43-1G-CFP in competition with NL43-3GYFP. Images in the mCherry, YFP, and CFP channels were collected every 30 minutes for 48-hours. Movies include 7 frames per second. CFP channel was normalized to account for detection differences.

Movie S3. Representative movie of NL43-1G-CFP in competition with NL43-1GYFP. Images in the mCherry, YFP, and CFP channels were collected every 30 minutes for 48-hours. Movies show 7 frames per second. CFP channel was corrected using correction factors derived from control samples like this example to normalize for detection sensitivity.

## Data Availability

All study data are included in the article and/or supporting information.
