# Supplementary material for: Role of RNA structural plasticity in modulating HIV-1 genome packaging and translation
Source: Proc Natl Acad Sci U S A. 2024 Aug 7;121(33):e2407400121. doi: 10.1073/pnas.2407400121 (PMC11331132; doi:10.1073/pnas.2407400121)
Supplement: Supplementary file 1 — Appendix 01 (PDF) [file pnas.2407400121.sapp.pdf]

Supporting Information for

Role of RNA structural plasticity in modulating HIV-1 genome packaging and translation

Saif Yasin[a,1], Sydney L. Lesko[b,c,1], Siarhei Kharytonchyk[d,1], Joshua D. Brown[a], Issac Chaudry[a], Samuel A. Geleta[a], Ndeh F. Tadzong[a], Mei Y. Zheng[a], Heer B. Patel[a], Gabriel Kengni Jr.[a], Emma Neubert[a], Jeanelle Mae C. Quiambao[a], Ghazal Becker[a], Frances Grace Ghinger[a], Sreeyasha Thapa[a], A'Lyssa Williams[a], Michelle H. Radov[a], Kellie X. Boehlert[a], Nele M. Hollmann[a,e], Karndeep Singh[a], James W. Bruce[b,c], Jan Marchant[a,*], Alice Telesnitsky[d,*], Nathan M. Sherer[b,c,*], and Michael F. Summers[a,e,*]

[a]Department of Chemistry and Biochemistry, University of Maryland, Baltimore Country, Baltimore, MD 21250; [b]McArdle Laboratory for Cancer Research, University of Wisconsin-Madison, Madison, WI 53705; [c]Institute for Molecular Virology, University of Wisconsin-Madison, Madison, WI 53705; [d]Department of Microbiology and Immunology, University of Michigan Medical School, Ann Arbor, MI 48109-5620; [e]Howard Hughes Medical Institute, University of Maryland, Baltimore County, Baltimore, MD 21250

[1]S.Y., S.L.L., and S.K. contributed equally to this work.

Corresponding Authors: Jan Marchant, Alice Telesnitsky, Nathan Sherer, Michael F. Summers

Email: janm@umbc.edu, ateles@umich.edu, nsherer@wisc.edu, summers@umbc.edu

This PDF file includes:

Supporting text
Figures S1 to S8
Tables S1 to S9
Legends for Movies S1 to S3
Legends for Datasets S1 to S4
SI References

Other supporting materials for this manuscript include the following:

Movies S1 to S3
Datasets S1 to S4

Supporting Information Text

Structural Conservation Analysis

Identification of 5′ polyA hairpin sequence. We extracted all complete HIV-1 genomes (20,439 depositions) present within the Los Alamos National Lab HIV compendium as of March 2, 2024. A majority of depositions were missing the 5′-LTR; therefore, only depositions containing the 5′-LTR were included for downstream analysis as determined by annotation of the *gag* start codon (1). To identify the hairpin within each deposition we developed a programmatic method to identify the most stable hairpin in the 5′-LTR that contained the hexameric polyadenylation signal (Fig. S2A). This approach would allow for a more thorough and robust assessment of 5′ polyA hairpin sequences through better defining the boundaries of the secondary structure element (2-4). For each deposition, we searched for the polyadenylation signal, AAUAAA, one of the most abundant cellular polyadenylation signals (5). The position of the signal is notated as position N_i, and an initial segment, F_i, is built around that point such that $F_i=[N_{i-75}, N_{i+75}]$. The assumption is that the true 5′ polyA hairpin sequence, F′, is a subsequence of F_i. We screened all possible subsequences that fall within F_i to identify F′ for each deposition. All subsequences within F_i were initially screened to be within a specific length range (35-50 nucleotides) and to have the AAUAAA signal near the center of the fragment such that at least 25% of the total fragment length was upstream and downstream of the signal. The secondary structures for all remaining subsequences were predicted using RNAfold from the ViennaRNA package (6). Fragments where more than 45% of residues were not base paired in the predicted secondary structure were removed. The remaining subsequence with the lowest predicted free energy is identified to be F′, the “true” 5′ polyA hairpin. If more than one instance of the AAUAAA signal is identified, all are considered, and the lowest energy structure is selected from the pool of predicted structures. A total of 1268 5′ polyA hairpin were identified, which falls within a similar range as other bioinformatic studies of the 5′ leader from the Los Alamos National Lab HIV compendium (7). A majority of identified hairpins were from subtype B (Fig. S2B).

RNA consensus structure alignment. A consensus secondary structure was inferred from all identified sequences and the 186 unique F′ sequences using the locARNA software tool (8-10). The positional frequencies of different base pair types in the helix were determined by aligning the consensus structure with each unique 5′ polyA hairpin identified. Alignment was performed using a modified Needleman-Wunsch algorithm with a scoring system that accounted for both the identity of nucleotides present in each base pair and the side of the helix each nucleotide was present on (example found in Fig. S2C-D) (11). Considering two base pairs (A_1, B_1) and (A_2, B_2), the scoring system was as follows:

Rule 1: If $A_1=A_2$ AND $B_1=B_2$, then the position received +1
Rule 2: If A_1 was found within (A_2, B_2) AND B_1 was found within (A_2, B_2) then the position received +0.75
Rule 3: If $A_1=A_2$ OR $B_1=B_2$, then the position received +0.5
Rule 4: If A_1 was found within (A_2, B_2) OR B_1 was found within (A_2, B_2) then the position received +0.25
Gaps received a -1 penalty.

In scoring alignments, a single rule with the highest score was applied to each base pair comparison and highest scoring alignment was taken as the true alignment. Following the alignment of each strain's 5′ polyA hairpin predicted structure to the consensus structure, the frequency of each base pair or bulge type that occurred at each position in the consensus structure was noted (Fig. 1D-E). Code for all steps described above is available at https://github.com/ichaudr1/hiv_polyA_struct_phylo_analysis.

Construction of the phylogenetic tree. We generated a phylogeny of 5′ polyA hairpins parsed using an alignment of the envelope gene via CLUSTALW (12-14). Representative strains were identified per subtype. For each subtype, the 5′ polyA seqeunce with the highest average similarity to all other 5′ polyA sequences from that subtype was determined to be the representative 5′ polyA for

that subtype. The percentage similarity, S, between F′ of two depositions was calculated as the proportion of sequence matches, m, to the total number of matches and mismatches, M, S=m/M. This metric was calculated in a pairwise manner for all depositions in each subtype. Strains with the highest similarity in each subtype were presented in the tree (Fig. S3). HIV-1^{MAL} (X04415) and HIV-1^{NL4-3} (KM390026) were also included as they were used in our subsequent biophysical studies. Additionally the HIV-1^{HXB2} (K03455) reference genome (15) was also included. A maximum likelihood tree was constructed and bootstrap support was inferred (n=1000) using PhyML (16). Predicted secondary structures and free energies were determined by the RNAfold program in the ViennaRNA software suite (6).

Free energy calculations for DIS, AUG, U5:AUG, and PolyA-U5:DIS 5′ leader elements. The sequence for each domain (DIS, AUG, U5:AUG, and PolyA-U5:DIS) was determined for each HIV-1 strain shown in Fig. S3. For each domain we utilized the boundaries established by previous NMR studies of the MAL leader monomer (AUG and PolyA-U5:DIS) and dimer (DIS and U5:AUG). The MAL leader (query) was aligned to each strain's full genome sequence (subject) using the Vector Builder online sequence alignment tool. For each domain of interest, we identified the region within the subject (other strains) that aligned to that of the query (MAL). This was then defined as the predicted domain sequence within the subject. The predicted secondary structure and RNA:RNA interaction free energies were then calculated using the ViennaRNA software suite (6) (Table 1 and Table S3). For comparison, we determined free energies of idealized RNA structures for MAL and NL4-3 strains that lacked noncanonical basepairs, bulges, and G:U wobbles (Table 1). Sequences identified are summarized in Dataset S2.

Preparation of un-capped and 5'-capped RNAs for in vitro biophysical studies

Preparation of DNA templates for in vitro RNA transcription. Plasmids containing leader sequences inserted within a Puc57 backbone were purchased from IDT DNA Technology. All leader sequences were preceded by the Top17 promoter sequence (5′-TAATACGACTCACTATA-3′) for RNA transcription. Mutations within the 5′ polyA region were generated using site directed mutagenesis (Q5® Site Directed Mutagenesis Kit, New England Biolabs). DNA templates were generated by PCR amplification (EconoTaq PLUS 2x Master Mix, Lucigen). A forward amplification primer 80 nucleotides upstream of the T7 promoter was used for all constructs. Reverse amplification primers had the first two 5′-residues 2′-O-methylated to reduce self-templated run-on (17). Plasmid and DNA template sequences were validated by Sanger sequencing (Eurofins Genomics). DNA templates were purified via sodium acetate and ethanol precipitation prior to transcription reactions. A complete list of plasmids and primers is shown in Tables S4, S5, and S6.

NTPs for in vitro transcription. Fully protiated rNTPs were purchased from Cayman Chemicals and resuspended to 100 mg/mL at pH 8.0. Perdeuterated and partially deuterated rNTPs were purchased from Cambridge Isotope Laboratories with the exception of GTP^{r} and ATP^{2} which were prepared in-house as previously described (17). Superscripts denote sites of protonation, while all other sites are deuterated, e.g., ATP^{2} = adenosines protiated at C2 only, GTP^{r}= guanosines protiated at all ribose positions.

Preparation of RNA by in vitro transcription. Uncapped RNAs were prepared through large scale in vitro transcription using T7 RNA polymerase as described previously (18). A 15 mL reaction contained ~1 mg of PCR amplified DNA template, 20 mM $MgCl_2$, 3 mM NTPs, 2 mM spermidine, 5 mM DTT, 20% (vol/vol) DMSO, 0.1% Triton X-100, 40 mM Tris·HCl (pH 9.0), and varying amounts T7 RNA polymerase (0.5-1 mg). Reaction conditions were optimized for each construct using small scale (30 μL) transcription reactions. Reactions were incubated for 6-10 hours at 37 °C, and then quenched with an EDTA solution (500 mM EDTA, pH 8.0) and were heated at 100°C on a heating block (VWR) for 5 minutes. Samples were snap cooled on ice for 5 minutes and then mixed with glycerol (final concentration, 6% [vol/vol]). Transcription reaction products were purified using 7.5 M urea polyacrylamide gels (19:1 acrylamide/bisacrylamide, SequaGel; National Diagnostics) at a constant power of 30 W for 16-24 hours. RNA was visualized by UV shadowing and then eluted from excised gel pieces using Elutrap electroelution systems (Whatman) at 130 V overnight. Eluted RNA was concentrated using Amicon Ultra centrifugal filters (Millipore). They were then rinsed

twice with 5 mL of 2 M high-purify NaCl followed by extensive desalting (8 x 5 mL millipore water). RNAs were evaluated for purity using small scale polyacrylamide gels post purification.

Preparation of in vitro capped RNA. Purified RNAs were 5′-capped using vaccinia virus capping enzyme, prepared in house as described (19). To maximize capping efficiency, RNAs were stored at -80°C to minimize hydrolysis of 5′-triphosphate. RNAs were boiled for 5 minutes and snap cooled for 5 minutes before capping. Capping reactions contained 20 µM RNA with 50 mM Tris base, 5 mM KCl, 1-3 mM $MgCl_2$, and 1 mM DTT (pH 8.0), 0.5 mM GTP, 0.1 mM S-adenosyl methionine, and varying amounts of vaccinia virus capping enzyme (20). Reaction conditions were optimized via small scale capping reactions (20 µL) of the RNA of interest in addition to separate reactions with a 35 nucleotide RNA that allows clear quantification of the addition of the cap residue by gel electrophoresis. Capping reactions were incubated for 1-2 hours at 37°C, quenched by the addition of 500 mM EDTA (pH 7.4) and then boiled for 5 minutes and snap cooled for 5 minutes. The capped RNA underwent gel purification, electroelution, and desalting using the same procedure described for RNAs prepared by in vitro transcription.

Preparation of RNA samples for DSC studies. DNA templates for the transcription of the 5′ polyA RNAs were prepared by annealing a 17 nucleotide T7 promoter sequence (Top17, 5′-TAATACGACTCACTATA-3′) to a reverse oligonucleotide purchased from IDT DNA Technology with the first two residues 2′-O-methyl modified to reduce nontemplated nucleotide addition by T7 RNA polymerase (17). The DNA oligos contained reverse complements of the sequences encoding the RNAs of interest (Table S7) as well as a Top17 binding sequence. Top17 (40 µL, 600 µM) was mixed with the reverse DNA oligo (80 µL, 200 µM). The mixture was then incubated in boiling water at 100 °C, and then slow cooled overnight. Millipore water (880 µL) was added to yield 1 mL of DNA template for direct use in transcription reactions to produce mg quantities of RNA. RNAs were produced and purified as described above.

Live Cell Imaging Analysis

Image correction methods. Images across three different channels with their respective excitation/emission filter sets, including YFP (490 to 510/520 to 550nm), CFP (325 to 375/435 to 485nm), and mCherry (565 to 590/590 to 650nm), were collected every 30 minutes for 48 hours. Three fields of view were acquired for each condition. To ensure reproducibility, two separate transfection experiments were performed for all conditions. All movies underwent background subtraction, baseline temporal drift correction, and vignetting correction using the BaSiC ImageJ plugin (10). HEK 293T cells exhibited limited mobility during the data collection period; therefore, regularization parameters for lambda flat and lambda dark were set to 3 as recommended.

Fluorescence quantification methods. Post correction, single cells were identified using Cellpose at each time point (Fig. S6A) (11). Cellpose masks were generated using the mCherry channel (Fig. 5C), with 14,685 cells identified across all experiments at the 30-hour timepoint. Cellpose masks with an area less than 100 px^2 were excluded to differentiate cells from image artifacts (48 total masks excluded). Cells with a raw integrated density (sum of pixel values) of zero in either YFP and CFP channels were also excluded to allow calculation of ($\log_2$(YFP /CFP)) ratios (0 total masks excluded at 30 hours, and no more than 64 masks excluded at any other timepoint). To control for co-transfection efficiency, we calculated the Pearson correlation coefficient between per-cell mean fluorescent intensity values of YFP and CFP for each condition (Fig. 4D, S6B-D), and excluded experiments from our analysis where the YFP:CFP correlation was relatively low (below 0.7).

Time dependent analysis methods. Plots of fluorescent intensity over time for each specific condition were generated through calculating the average mean fluorescence intensity (MFI) across all cells for each time point for both CFP and YFP independently (Fig. 5A-B). As shown in Fig. 5A-B, the virus exhibited linear increases to Gag-CFP/YFP fluorescence intensity between 12

and 36 hours post-transfection, so we selected the 30-hour time point for detailed statistical analysis (Fig. 5D, Dataset S3 and S4).

YFP:CFP ratio calculation. YFP:CFP ratios were calculated for each cell at the 30-hour time point (Fig. 5D). To treat ratio values symmetrically and allow for simpler error analysis, log-ratios were calculated using log base 2 ($\log_2$(YFP /CFP)) (12). Log-ratios across all regions of interest for a single biological replicate were averaged. To control for any differences in Gag-CFP and Gag-YFP fluorescence detection that may reflect differences in detection parameters across experiments, we corrected each dataset by normalizing values across channels (13). To this end, six control samples where identical promoter and leader sequences were present in both YFP and CFP plasmids were measured for each independent transfection experiment (two total independent transfection experiment on separate plates measured for each condition). The average control log-ratio for each plate (Fig. S7) was used to correct experimental ratios for each cell from the same plate. 95% confidence intervals of the standard error of the mean were calculated for both experimental and control sample log ratios across all biological replicates, reporting cell to cell variation within each condition (Fig. 5C). Additionally, ratios were calculated for each sample condition at every time-point between 24-48 hours to compare trends across different timepoints. All samples were corrected using control ratios measured at their respective timepoint (Fig. S8).

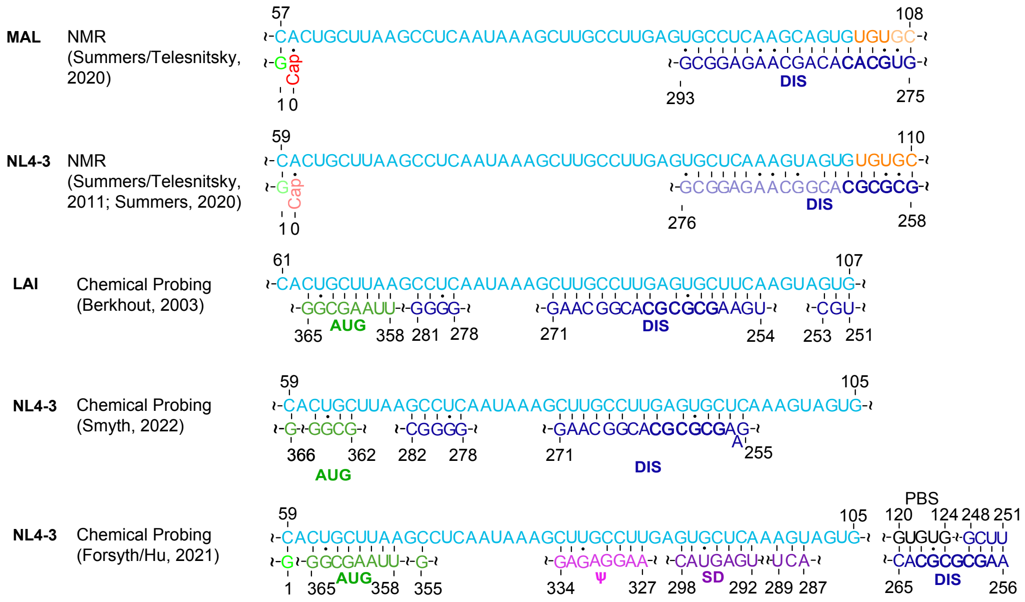

Fig. S1. 5′ polyA structures exhibit variation across different monomeric leader structural models. Different models are shown from top down. (Top) Nuclear Magnetic Resonance (NMR) based structure for the ^{Cap}3G leader of HIV-1$_{\mathrm{MAL}}$ strain (nts: 1-371) (21). The 5′ polyA interacts with residues of TAR and the DIS, but largely remains unstructured. (Second) NMR based model for 5′ polyA structure in the HIV-1$_{\mathrm{NL4\text{-}3}}$ strain in a 3G monomeric leader based upon data from a 2G leader mutated to favor the monomer conformation (nts: 2-357) (22). Residues predicted to interact but not directly observed by NMR are shown transparent. (Third) Berkhout long distance interaction (LDI) model based upon chemical probing data of the HIV-1$_{\mathrm{LAI}}$ strain (nts: 2-369) 2G monomeric leader (23). The 5′ polyA forms base pairs with the DIS and Gag coding sequence. (Fourth) Forsyth-Hu Model (nts: 1-401) shows the 5′ polyA interacting with the first residue of TAR, ψ, SD, and the Gag coding sequence using chemical probing of the HIV-1$_{\mathrm{NL4\text{-}3}}$ strain in a 3G monomeric leader. (Bottom) Smyth Model (nts: 1-381) shows the 5′ polyA interacting with the DIS and Gag coding sequence using chemical probing of the HIV-1$_{\mathrm{NL4\text{-}3}}$ strain in a 3G monomeric leader. Numbering for all constructs is based upon the first guanosine in a 3G RNA being residue 1. The DIS palindrome is bolded in all constructs.

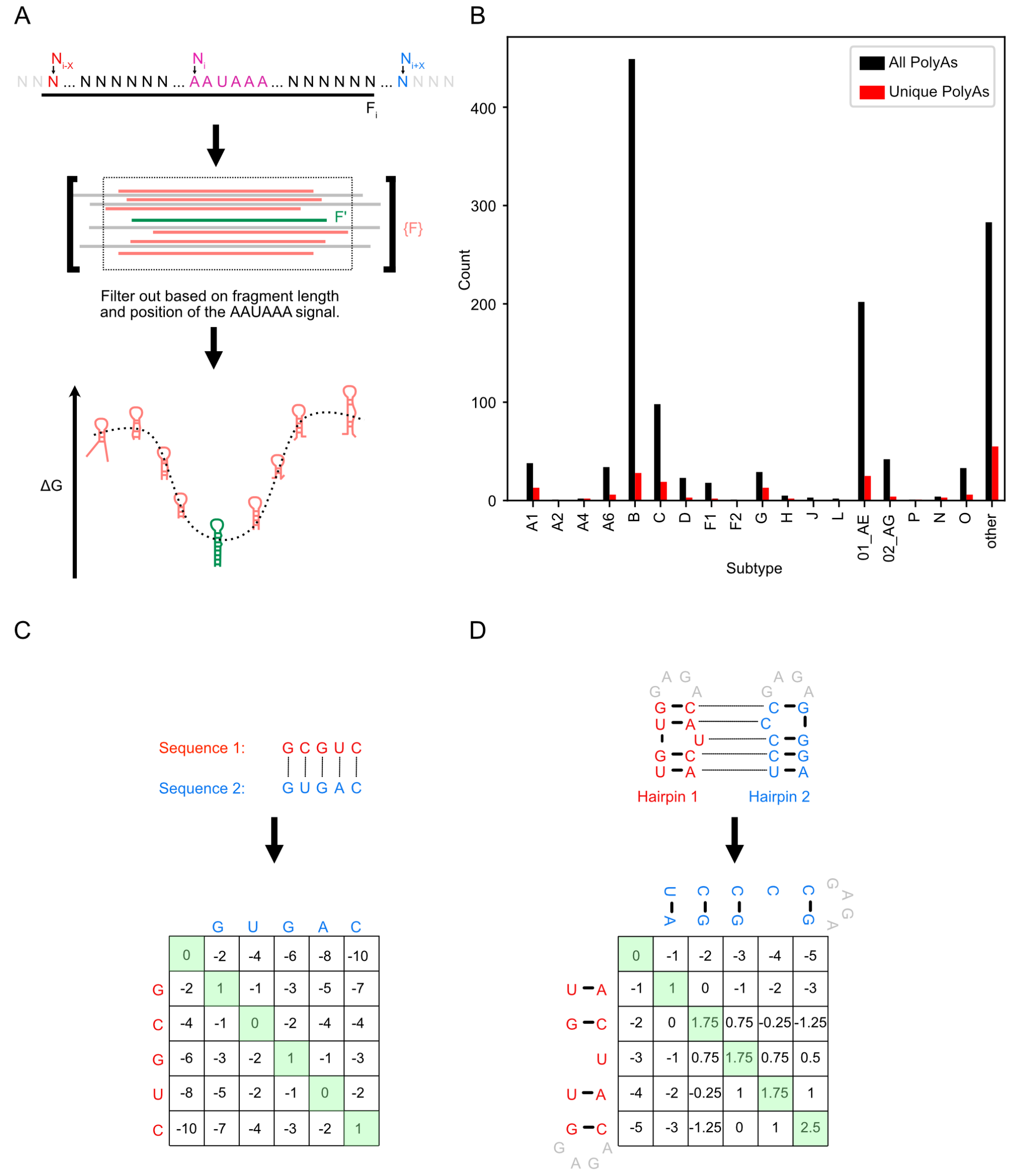

Fig. S2. Method for bioinformatics analysis. (A) An initial fragment, F_i, is selected, which is rooted at the AAUAAA polyadenylation signal and extends 75 nucleotides upstream and downstream the first adenosine residue. Each sub-sequence, F_i, within F is screened based on length and polyadenylation signal position. All remaining fragments undergo RNA secondary structure prediction and free energy predictions via ViennaRNA (6). The fragment predicted to form the lowest free energy structure is selected as the 5′ polyA for that deposition. (B) HIV-1 5′ polyA counts by subtype. 5′ polyA hairpin sequences were identified within 1268 depositions of the Los Alamos National Laboratory compendium (1). The numbers of 5′ polyA hairpins and unique 5′ polyA hairpins identified within each subtype are shown, including main subtypes (A1, A2, A4, A6, B, C, D, F1, F2, G, H, J, L, 01_AE, 02_AG, P, N, O) and rarer recombinant subtypes (other). (C) A schematic showing the dynamic programming setup for a traditional Needleman-Wunsch algorithm for aligning sequences (11). (D) The analogous setup of our modified Needleman-Wunsch algorithm with our scoring system that has been adapted to align helical positions instead of sequence positions.

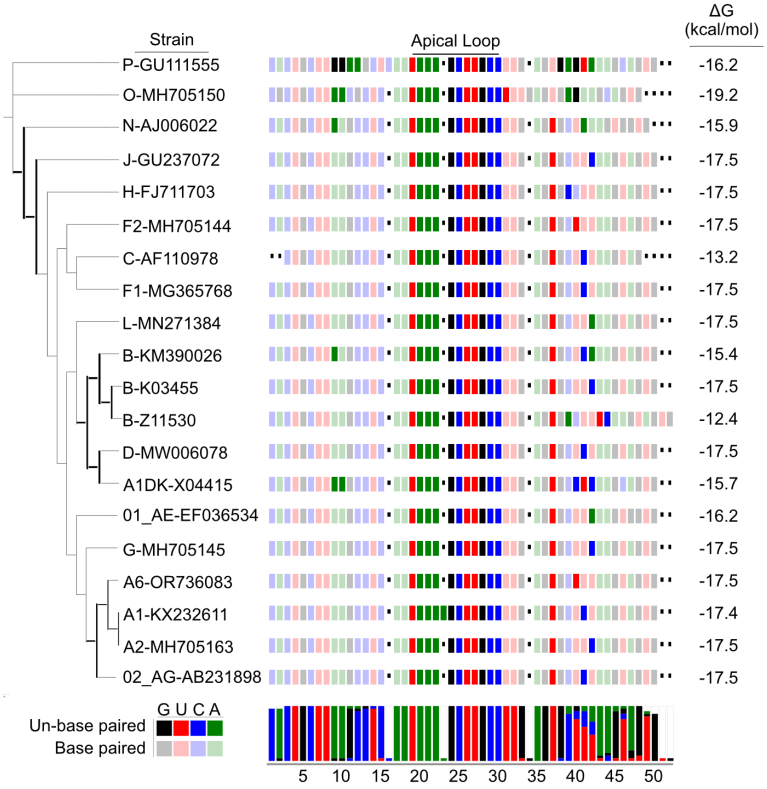

Fig. S3. Inferred phylogeny for representative 5′ polyA sequences. An inferred phylogeny of representative HIV-1 strains from each major subtype within the Los Alamos National Laboratory compendium (1). HIV-1^{MAL} (X04415), HIV-1^{NL4-3} (KM390026), and the reference genomes HIV-1^{HXB2} (K03455) were also included for reference. The phylogeny was built with bootstrapping (n=1000, branch width indicates branch support) based upon a multiple sequence alignment of the viral envelope protein sequence. The tree is annotated with a multiple sequence alignment of the 5′ polyA hairpin as colored blocks where solid blocks indicate residues that are un-base paired and transparent blocks indicate residues that are base paired in the predicted secondary structure by ViennaRNA (6), with predicted free energies reported. Positional nucleotide frequencies are represented as a bar graph where white space in the bars represents gaps in the alignment.

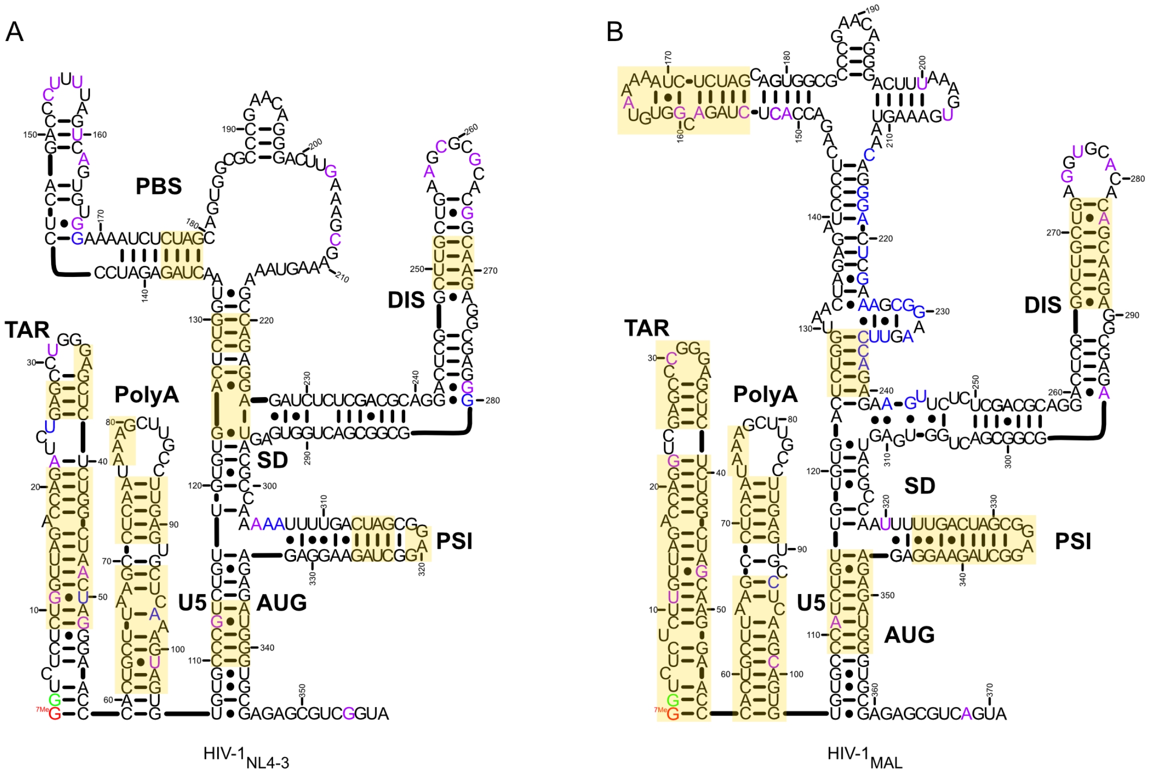

Fig. S4. NMR derived 5′ leader secondary structure for HIV-1_{NL4-3} (A) and HIV-1_{MAL} (B). Substitutions with respect to the other leader are shown in purple. Additions with respect to the other leader are shown in blue. Red indicates the 5′ cap. Green indicates the initial guanosine residues. Yellow boxes indicate regions supported to form by NOESY results as described previously (17, 24).

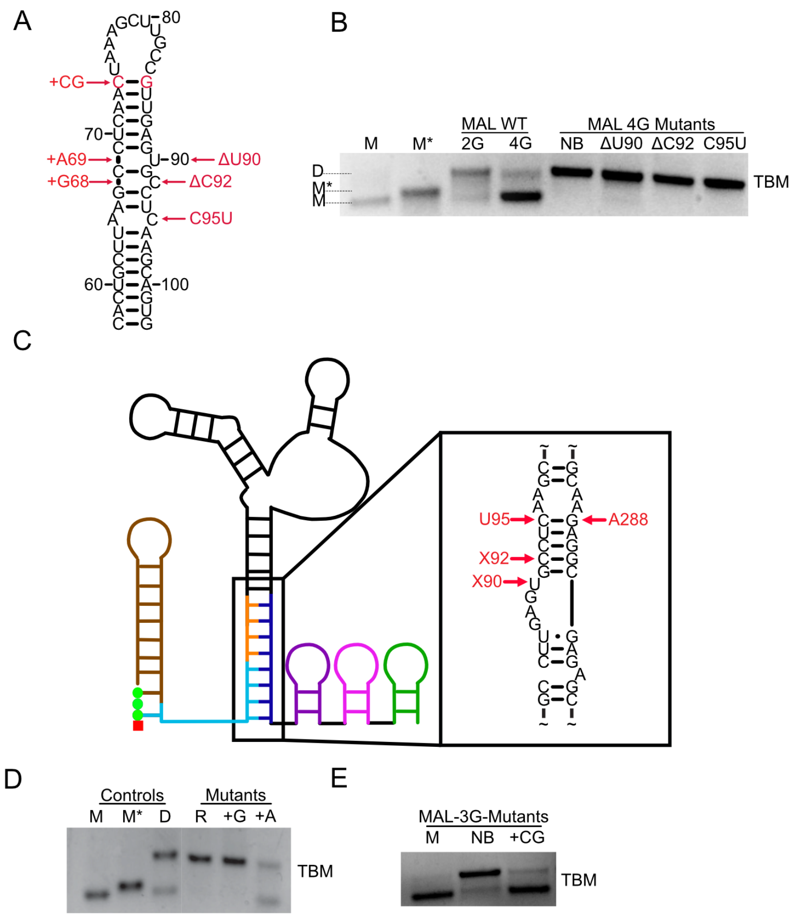

Fig. S5. In vitro dimerization assays of 5′ polyA mutant leaders. (A) HIV-1_{MAL} 5′ polyA hairpin secondary structure with all mutations of interest denoted. (B) In vitro dimerization assay of 4G leaders where a single 5′ polyA bulge was stabilized or deleted. Controls present include a monomer (M), a monomer in the dimer promoting conformation (M*), the MAL-2G Leader, the MAL-4G Leader, and the MAL-4G-NB Leader with all three bulges mutated. (C) Cartoon of proposed monomeric leader structure (24) to highlight the potential role these bulge residues may play in the monomer. (D) In vitro dimerization assay of mutants to assess whether monomer destabilization contributed to the equilibrium shift upon mutating bulges (R=C95U-G288A, +G=+G68, +A=+A69). (E) In vitro dimerization assay assessing the role of an additional base pair (+CG) at the top of the 5′ polyA hairpin.

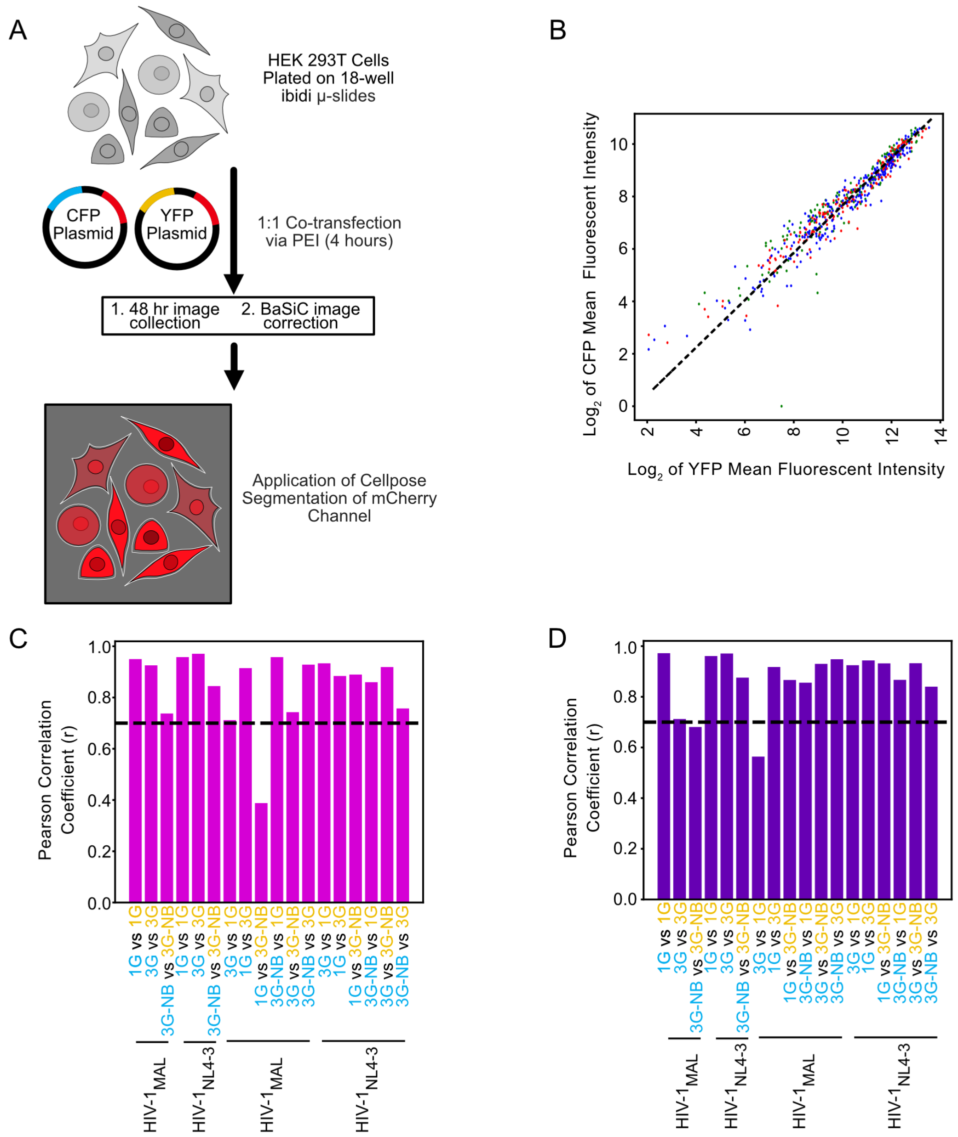

Figure S6. Competitive translation assay validation. (A) Human embryonic kidney (HEK) 293T Cells were plated and co-transfected to express Gag-CFP/mCherry and Gag-YFP/mCherry reporter viruses modified to encode the indicated U3 (1G vs. 3G) and 5′-leader sequences (WT vs NB). Cells were subjected to live cell imaging for 48h with images collected every 30 minutes. Images were corrected for shading and temporal drift using BaSiC. Cellpose segmentation was applied to each image using the mCherry signal as a fluid phase marker to generate masks for co-transfected cells, which were then applied individually to both the YFP and CFP channels. We then calculated single cell YFP:CFP log ratios. (B) Example plot of individual cell $\log_2$(YFP MFI) versus $\log_2$(CFP MFI) showing strong correlation of single cell Gag-CFP and Gag-YFP expression levels. Linear trendline from Fig. 4D is plotted. (C, D) Calculated Pearson correlation coefficients (r) of YFP MFI versus CFP MFI for each well from two separate transfections at 30 hours post transfection (C and D respectively). Dashed line at 0.7 shows our threshold value for inclusion in downstream analysis.

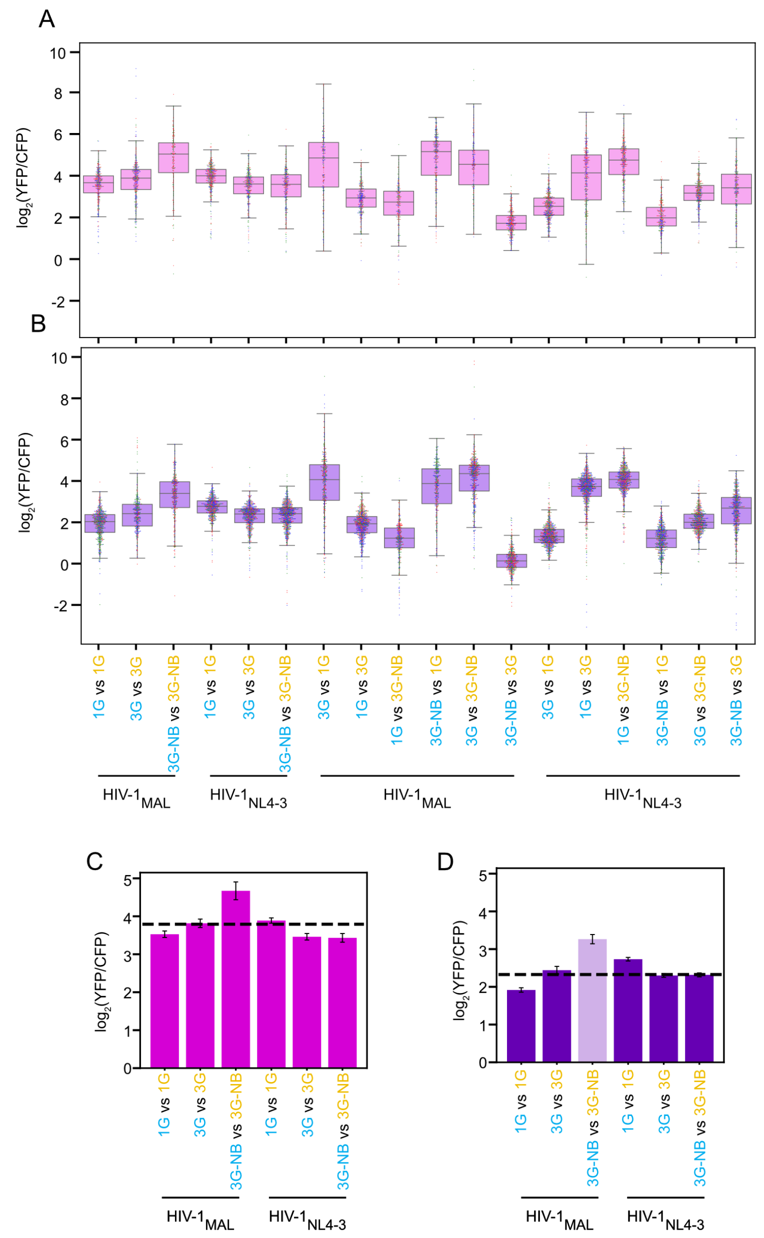

Figure S7. Determination of a correction factor to account for differences to YFP and CFP detection across biological replicates (different days and/or microscopes). (A, B) Box and whisker plots of average per cell log_2(YFP/CFP) ratio for each sample across two separate transfections (A and B, shown in pink and purple respectively). Boxes represent the first to third quartile range with the median marked in the middle. Whiskers show the range of 1.5 times the interquartile range. Points depict individual cells, which are colored based upon which region of interest the cells originate from as three were collected for each well. (C, D) Average log_2(YFP/CFP) ratios for control samples from two separate transfections where the identical leader sequences were in competition. The dotted line represents the average value, which was used to correct all data collected from the same hardware set up from two different transfections (C and D, shown in pink and purple respectively). Error bars show the 95% confidence interval of the standard error of the mean across all cells. The light purple bar indicates that this ratio was not included in the average due to a correlation coefficient below threshold of 0.7 (Fig. S6).

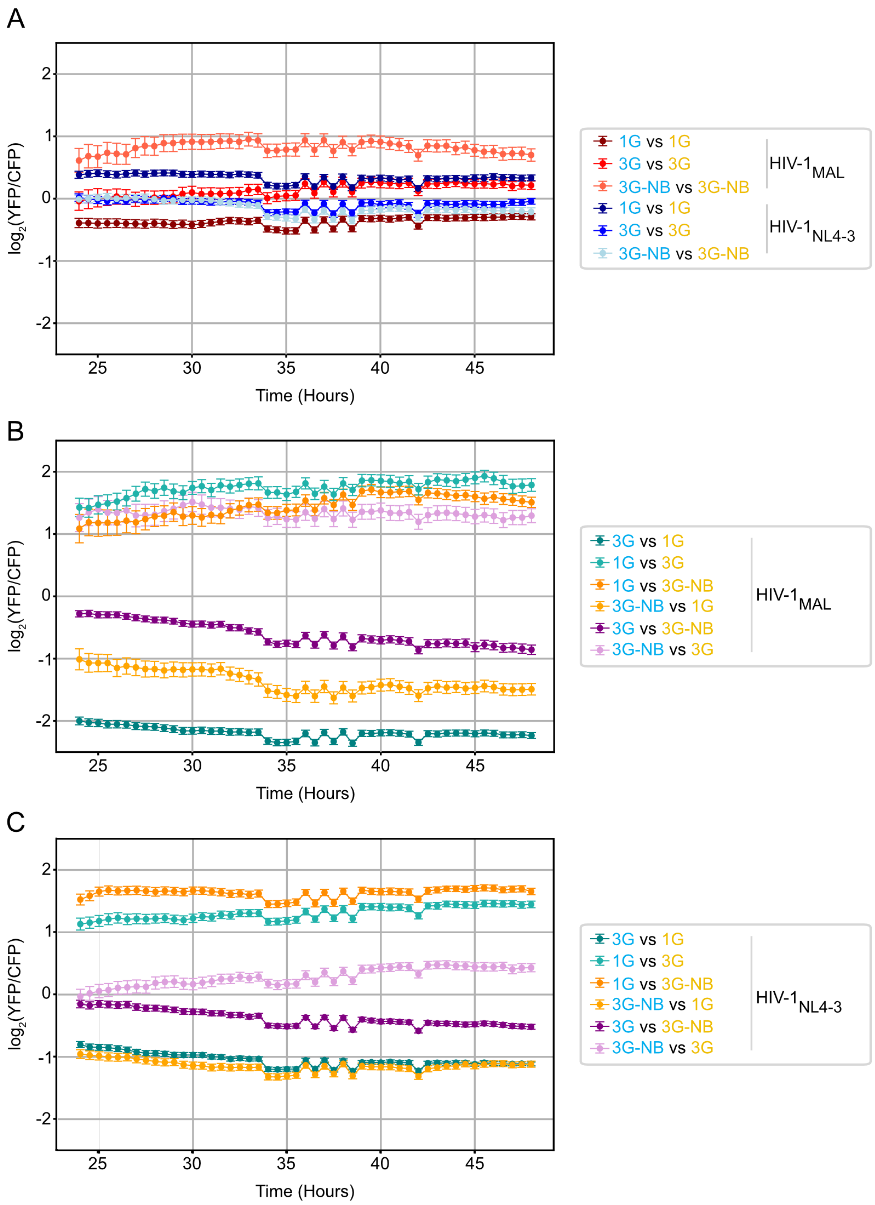

Figure S8. log_2(YFP/CFP) ratio over time. (A, B, C) Plots of the average log_2(YFP/CFP) ratio across all cells at each indicated timepoint for controls (A), HIV-1$_{MAL}$ experimental conditions (B), and HIV-1$_{NL4\text{-}3}$ experimental conditions (C). All cells were corrected for differences in YFP and CFP sensitivity using correction factors derived from the matching timepoint.

Table S1. Differential scanning calorimetry fitting parameters of 5′ polyA hairpins

5′ polyA Hairpin	Aw ± 95% CI	T_m ± 95% CI (°C)	ΔH ± 95% CI (kJ/mol)
HIV-1$_{MAL}$ Wild type	1.409 ± 0.108	67.63 ± 0.122	511.7 ± 23.91
HIV-1$_{MAL}$ No Bulge (NB)	0.773 ± 0.051	83.16 ± 0.083	796.6 ± 30.59
HIV-1$_{MAL}$ ΔU90	0.759 +/- 0.071	74.61 +/- 0.104	719.2 +/- 42.97
HIV-1$_{MAL}$ ΔC92	0.950 ± 0.080	71.25 ± 0.099	668.4 ± 33.63
HIV-1$_{MAL}$ C95U	0.751 ± 0.083	71.52 ± 0.128	712.5 ± 43.76
HIV-1$_{MAL}$ +A69	0.888 ± 0.077	73.63 ± 0.098	753.1 ± 39.66
HIV-1$_{MAL}$ +G68	0.946 ± 0.059	74.71 ± 0.079	722.0 ± 26.85

Table S2. Isothermal titration calorimetry fitted stoichiometries and K_d's for NC binding to dimeric RNAs

Leader Construct	N ± St. Dev.	K_d ± St. Dev (μM)
HIV-1_{MAL} 4G-NB	43.05 ± 0.35	3.11 ± 0.73
HIV-1_{MAL} 2G	45.05 ± 5.02	1.00 ± 0.03
HIV-1_{NL4-3} 4G-NB	38.65 ± 9.12	2.24 ± 1.94
HIV-1_{NL4-3} 2G	34.7 ± 3.81	2.48 ± 0.28

Table S3. Predicted free energies of 5'-leader helical elements for representative strains of HIV-1

Strain Subtype and Accession	PolyA Hairpin	DIS Hairpin	AUG Hairpin	U5:AUG Helix	PolyA-U5:DIS Helix
P-GU111555	-16.2	-17.0	-3.1	-13.9	-9.2
O-MH705150	-19.2	-16.8	-4.3	-13.6	-4.9
N-AJ006022	-15.9	-15.3	-3.1	-18.9	-13.8
J-GU237072	-17.5	-13.0	-3.1	-16.6	-15.4
H-FJ711703	-17.5	-14.1	-3.1	-16.6	-18.1
F2-MH705144	-17.5	-13.0	-3.1	-16.6	-13.8
C-AF110978	-13.2	-15.4	-3.1	-16.6	-4.8
F1-MG365768	-17.5	-14.5	-3.1	-16.6	-4.8
L-MN271384	-17.5	-13.9	-3.1	-16.6	-12.4
B-KM390026	-15.4	-13.2	-4.7	-16.6	-13.3
B-K03455	-17.5	-13.2	-3.1	-16.6	-17.9
B-Z11530	-12.4	-13.9	-3.1	-13.0	-11.3
D-MW006078	-17.5	-13.5	-3.1	-16.6	-13.8
A1DK-X04415	-15.7	-13.0	-3.1	-17.0	-19.6
01_AE-EF036534	-16.2	-13.0	-3.1	-16.7	-15.3
G-MH705145	-17.5	-12.3	-3.1	-16.6	-16.4
A6-OR736083	-17.5	-13.0	-4.7	-15.6	-13.9
A1-KX232611	-17.4	-13.2	-3.1	-16.6	-11.1
A2-MH705163	-17.5	-12.0	-3.1	-16.6	-13.0
02_AG-AB231898	-17.5	-11.7	-3.1	-16.6	-12.2
Average	-16.7	-13.8	-3.3	-16.3	-12.7
Standard Deviation	-1.6	-1.4	-0.5	-1.3	-4.2

* Sequences and strains shown in Fig. S3. Values reported in kCal/mol, calculated using ViennaRNA (6).

Table S4. Plasmids used for in vitro RNA synthesis in this study

Plasmid	Description
HIV-1_{MAL} 5′ leader	WT HIV-1_{MAL} 5′-Leader sequence (ID:X04415.1, nt 1-370). NCBI entry begins with two guanosines. Plasmids with three and four guanosines were also generated. For consistency residue number is based on the first guanosine in a 3G being residue 1.
HIV-1_{NL4-3} 5′ leader	WT HIV-1_{NL4-3} 5′-Leader sequence (ID:MN685352.1, nt 1-356). NCBI entry begins with two guanosines. A plasmid with four guanosines was also generated. Residue numbering as described for HIV-1_{MAL}.
HIV-1_{MAL} 5′ leader-NB*	HIV-1_{MAL} 5′-Leader (nt 1-370) ΔT90, ΔC92, C95T for 1G, 2G, 3G, and 4G. Constructs with individual mutations were also constructed for 4G: ΔT90, ΔC92, C95T, +G68, +A69, C95T-G288A.
HIV-1_{NL4-3} 5′ leader-NB*	HIV-1_{NL4-3} 5′-Leader (nt 1-356) ΔT92, C96T, ΔA98 for 1G, 2G, 3G, and 4G.
Locked Monomer	HIV-1_{MAL} 5′-Leader (4G) 96-AAGCA-100 replaced for 96-TTACT-100 and C285T
Locked Monomer*	HIV-1_{MAL} 5′-Leader (2G) 271-AGGTGCACA-279 replaced from 271-GAGA-274

*Δ=deletion of residue, +=addition of residue

Table S5. Primers used for Site Directed Mutagenesis for in vitro RNA synthesis designed using NEBaseChanger

Name	Description	Forward Primer	Reverse Primer
MAL 3G 5′ leader	Generate a 3G MAL 5′-Leader	5′-GGGTCTCTCTTGTTAGACC-3′	5′-TATAGTGAGTCGTATTAGG-3′
MAL 4G 5′ leader	Generate a 4G MAL 5′-Leader	5′-GGGGTCTCTCTTGTTAGACC-3′	5′-TATAGTGAGTCGTATTAGG-3′
NL4-3 4G 5′ leader	Generate a 4G NL43 5′-Leader	5′-GGGGTCTCTCTGGTTAGACC-3′	5′-TATAGTGAGTCGTATTAGAATTC-3′
MAL 5′ leader NB	Stabilize or delete bulges in the 5′ polyA region	5′-TTGCCTTGAGGCTTAAGCAGTGTG-3′	5′-GCTTTATTGAGGCTTAAG-3′
NL43 5′ leader NB	Stabilized or delete bulges in the 5′ polyA region	5′-TAAGTAGTGTGTGCCCGTCT-3′	5′-AGCCTCAAGGCAAGCTTTATTGAG-3′
MAL 5′ leader ∆T90	Delete a single bulge in 5′ polyA region	5′-GCCTCAAGCAGTGTGTGC-3′	5′-CTCAAGGCAAGCTTTATTGAGG-3′
MAL 5′ leader ∆C92	Delete a single bulge in 5′ polyA region	5′-CTCAAGCAGTGTGTGCCC-3′	5′-CACTCAAGGCAAGCTTTATTG-3′
MAL 5′ leader C95T	Stabilize a single bulge in 5′ polyA region	5′-TTGAGTGCCTTAAGCAGTGTG-3′	5′-GGCAAGCTTTATTGAGGC-3′
MAL 5′ leader G288A	Mutation in DIS region to understand role of 5′ polyA bulges	5′-CACACAGCAAAAGGCGAGAGC-3′	5′-CACCTCAGCAAGCCGAGTC-3′
Locked Monomer Mutation 1	Mutation in 5′ PolyA-U5 region to force a monomer	5′-TTGAGTGCTCTTAGTCGTGTGTGCCC-3′	5′-GGCAAGCTTTATTGAGGC-3′
Locked Monomer Mutation 2	Mutation in DIS region to force a monomer	5′-CGCGCACGGCTAAGGGCGAGGGGC-3′	5′-CTTCAGCAAGCCGAGTCCTG-3′
Locked Monomer*	Mutation in DIS region to force a monomer in the dimeric conformation	5′-GACAGCAAGAGGCGAGAGCG-3′	5′-TCCAGCAAGCCGAGTCCTGC-3′

Table S6. Primers used for the PCR based preparation of DNA templates for RNA transcription

Name	Description	Sequence
Universal Forward Primer	Forward Primer used for all reactions	5′-GGGATGTGCTGCAAGGCGATTAAGTTGGG-3′
HIV-1_{MAL} Reverse Primer	Reverse Primer used for all HIV-1_{MAL} constructs	5′-mUmACTGACGCTCTCGCACCCATCTCT-3′
HIV-1_{NL4-3} Reverse Primer	Reverse Primer used for all HIV-1_{NL4-3} constructs	5′-mUmACCGACGCTCTCGCACCCATCTCTC-3′

*m denotes the incorporation of 2′-O-methyl RNA bases

Table S7. 5′ polyA hairpin synthesized via in vitro transcription for DSC experiments

Hairpin Construct	Sequence
HIV-1$_{MAL}$ Wild type	5′-**GG**CACUGCUUAAGCCUCAAUAAAGCUUGCCUUGAGUGCCUCAAGCAGUG**CC**-3′
HIV-1$_{MAL}$ No Bulge (NB)	5′-**GG**CACUGCUUAAGCCUCAAUAAAGCUUGCCUUGAGGCUUAAGCAGUG**CC**-3′
HIV-1$_{MAL}$ ∆U90	5′-**GG**CACUGCUUAAGCCUCAAUAAAGCUUGCCUUGAGGCCUCAAGCAGUG**CC**-3′
HIV-1$_{MAL}$ ∆C92	5′-**GG**CACUGCUUAAGCCUCAAUAAAGCUUGCCUUGAGUGCUCAAGCAGUG**CC**-3′
HIV-1$_{MAL}$ C95U	5′-**GG**CACUGCUUAAGCCUCAAUAAAGCUUGCCUUGAGUGCCUUAAGCAGUG**CC**-3′
HIV-1$_{MAL}$ +A69	5′-**GG**CACUGCUUAAGCACUCAAUAAAGCUUGCCUUGAGUGCCUCAAGCAGUG**CC**-3′
HIV-1$_{MAL}$ +G68	5′-**GG**CACUGCUUAAGGCCUCAAUAAAGCUUGCCUUGAGUGCCUCAAGCAGUG**CC**-3′

*Bolded residues were non-native nucleotides included to improve transcription yields.

Table S8. Primers used for Overlapping PCR Based Mutagenesis

Name	Description	Forward Primer	Reverse Primer
HIV-1$_{MAL}$ 1G	Generate a plasmid that will produce WT/NB MAL ^{Cap}1G RNA in cells	5′-CAGATGCTACATATAAGCAGCTGCTTTTTGCCTGTACTTCGTCTCTCTTGTTAGACCA -3′	5′-ACCGAATTTTTCCCATTTATCTAATTCTCCCCCGCTTAATACTGACGCTCTCGCACCCA-3′
HIV-1$_{MAL}$ 3G	Generate a plasmid that will produce WT/NB MAL ^{Cap}3G RNA in cells	5′-CAGATGCTACATATAAGCAGCTGCTTTTTGCCTGTACTTCGGGTCTCTCTTGTTAGACCA -3′	Identical to MAL 1G Reverse Primer
HIV-1$_{NL4-3}$ 1G	Generate a plasmid that will produce WT/NB NL4-3 ^{Cap}1G RNA in cells	5′-CAGATGCTACATATAAGCAGCTGCTTTTTGCCTGTACTTCGTCTCTCTGGTTAGACCAGA-3′	5′-ACCGAATTTTTCCCATTTATCTAATTCTCCCCCGCTTAATACCGACGCTCTCGCACCCA-3′
HIV-1$_{NL4-3}$ 3G	Generate a plasmid that will produce WT/NB NL4-3 ^{Cap}3G RNA in cells	5′-CAGATGCTACATATAAGCAGCTGCTTTTTGCCTGTACTTCGGGTCTCTCTGGTTAGACCA -3′	Identical to NL4-3 1G Reverse Primer

*Underlined sequence is complementary to Puc57 vectors from Table S4 to get 5′ leader wildtype and no bulge sequences for insertion into dual reporter system

Table S9. Primers and Gene Block for RT-qPCR quantification of total HIV RNA transcript

Name	Description	Sequence
HIV-Transcript Forward	Forward primer for total HIV-1 transcript	5′-CAGATGCTGCATATAAGCAGCTG-3′
HIV-Transcript Reverse	Reverse primer for total HIV-1 transcript	5′-TTTTTTTTTTTTTTTTTTTTTTGAAGC ACTC-3′
HIV-Transcript Probe	Molecular Beacon probe for total HIV-1 transcript with fam (5′), zen (internal), and iowa black (3′) labeling	5′-CCTGTACTGGGTCTCTCTGG-3′
HIV-Transcript Gene Block	5′- ATAGGATCCCAGGTATCCTTTGAGCCAATTCCCATACATTATTGTGCCC CGGCTGGTTTTGCGATTCTAAAATGTAATAATAAGACGTTCAATGGAACAGG ACCATGTACAAATGTCAGCACAGTACAATGTACACATGGAATTAGGCCAGTA GGAAGGGCACATAGCCAGAAATTGCAGGGCCCCTAGGAAAAAGGGCTGTT GGAAATGTGGAAAGGAAGGACACCAAATGAAAGATTGTACTGAGAGACAGG CGAGTTTGTCAATACCCCTCCCTTAGTGAAGTTATGGTACCAGTTAGAGAAA GAACCCATAATAGGAGCAGAAACTTTCTATGTAGATGGGGCAGCCAATAGG GAAACTAAATTAGGAAAAGCAGGATATGTAACTGACAGAGGCCTCCCATCAG TGGACAAATTAGATGTTCATCAAATATTACTGGGCTGCTATTAACAAGAGATG GTGGTAATAACAACAATGGGTCCGAGATCTTCAGAGAGGCCAATAAAGGAG AGAACACCAGCTTGTTACACCCTGTGAGCCTGCATGGAATGGATGACCCTG AGAGAGAAGTGTTAGAGTGGAGGTTTGACAGCCAGATGCTGCATATAAGCA GCTGCTTTTTGCCTGTACTGGGTCTCTCTGGTTAGACCAGATCTGAGCCTGG GAGCTCTCTGGCTAACTAGGGAACCCACTGCTTAAGCCTCAATAAAGCTTGC CTTGAGTGCTTCAAAAAAAAAAAAAAAACTTTGTCAAGCTCATTTCCTGGTATG ACAACGAATTTGGCTACAGCAACAGGGTGGTGGACCTCATGGCCCACATGG CCTCCAAGGAGTAAGACCCCTGGACCACCAGCCCCAGCAAGAGCACAAGA GGAAGACTCGAGATA-3′	

Movies and Datasets

Movie S1 (separate file). Representative movie of NL43-3G-CFP in competition with NL43-1G-YFP. Images in the mCherry, YFP, and CFP channels were collected every 30 minutes for 48-hours. Movies include 7 frames per second. CFP channel was normalized to account for detection differences.

Movie S2 (separate file). Representative movie of NL43-1G-CFP in competition with NL43-3G-YFP. Images in the mCherry, YFP, and CFP channels were collected every 30 minutes for 48-hours. Movies include 7 frames per second. CFP channel was normalized to account for detection differences.

Movie S3 (separate file). Representative movie of NL43-1G-CFP in competition with NL43-1G-YFP. Images in the mCherry, YFP, and CFP channels were collected every 30 minutes for 48-hours. Movies show 7 frames per second. CFP channel was corrected using correction factors derived from control samples like this example to normalize for detection sensitivity.

Dataset S1 (separate file). Statistics for individual ITC experiments and fittings.

Dataset S2 (separate file). Sequences utilized for prediction of free energies of 5′ polyA, DIS, and AUG hairpins as well as U5:AUG and polyA-U5:DIS helices.

Dataset S3 (separate file). Individual cell data for all cells identified within translation assay experiments.

Dataset S4 (separate file). Statistical data for ratios for all translation assay conditions.

SI References

1. Anonymous (2016) Los Alamos HIV sequence compendium 2016.
2. R. Sabarinathan, C. Anthon, J. Gorodkin, S. E. Seemann, Multiple sequence alignments enhance boundary definition of RNA structures. *Genes* **9**, 604 (2018).
3. B. Zhang, D. T. Yehdego, K. L. Johnson, M.-Y. Leung, M. Taufer, Enhancement of accuracy and efficiency for RNA secondary structure prediction by sequence segmentation and MapReduce. *BMC Structural Biology* **13**, 1-24 (2013).
4. R. Sabarinathan *et al.*, RNA snp: efficient detection of local RNA secondary structure changes induced by SNP s. *Human mutation* **34**, 546-556 (2013).
5. B. Tian, J. Hu, H. Zhang, C. S. Lutz, A large-scale analysis of mRNA polyadenylation of human and mouse genes. *Nucleic acids research* **33**, 201-212 (2005).
6. R. Lorenz *et al.*, ViennaRNA Package 2.0. *Algorithms for Molecular Biology* **6**, 26 (2011).
7. M. I. Zarudnaya, A. L. Potyahaylo, I. Kolomiets, D. M. Hovorun, Phylogenetic study on structural elements of HIV-1 poly (A) region. 1. PolyA and DSE hairpins. *Biopolymers and cell*, 454-462 (2013).
8. S. Will, I. L. Joshi T Fau - Hofacker, P. F. Hofacker Il Fau - Stadler, R. Stadler Pf Fau - Backofen, R. Backofen, LocARNA-P: accurate boundary prediction and improved detection of structural RNAs.
9. S. Will, I. L. Reiche K Fau - Hofacker, P. F. Hofacker Il Fau - Stadler, R. Stadler Pf Fau - Backofen, R. Backofen, Inferring noncoding RNA families and classes by means of genome-scale structure-based clustering.
10. M. Raden *et al.*, Freiburg RNA tools: a central online resource for RNA-focused research and teaching. *Nucleic Acids Research* **46**, W25-W29 (2018).
11. C. D. Needleman Sb Fau - Wunsch, C. D. Wunsch, A general method applicable to the search for similarities in the amino acid sequence of two proteins.
12. L. Perrin, S. Kaiser L Fau - Yerly, S. Yerly, Travel and the spread of HIV-1 genetic variants.
13. C. Pasquier *et al.*, HIV-1 subtyping using phylogenetic analysis of pol gene sequences.
14. J. D. Thompson, D. G. Higgins, T. J. Gibson, CLUSTAL W: improving the sensitivity of progressive multiple sequence alignment through sequence weighting, position-specific gap penalties and weight matrix choice. *Nucleic Acids Res* **22**, 4673-4680 (1994).
15. C. Wymant *et al.*, Easy and accurate reconstruction of whole HIV genomes from short-read sequence data. *biorxiv*, 092916 (2016).
16. S. Guindon *et al.*, New algorithms and methods to estimate maximum-likelihood phylogenies: assessing the performance of PhyML 3.0. *Systematic biology* **59**, 307-321 (2010).
17. S. C. Keane *et al.*, NMR detection of intermolecular interaction sites in the dimeric 5'-leader of the HIV-1 genome. *Proc Natl Acad Sci U S A* **113**, 13033-13038 (2016).
18. J. F. Milligan, O. C. Uhlenbeck, Synthesis of small RNAs using T7 RNA polymerase. *Meth. Enzymol.* **180**, 51-62 (1989).
19. P. Ding *et al.*, 5'-Cap sequestration is an essential determinant of HIV-1 genome packaging. *Proc Natl Acad Sci U S A* **118**, 1-8 (2021).
20. A. L. Fuchs, A. Neu, R. Sprangers, A general method for rapid and cost-efficient large-scale production of 5' capped RNA. *RNA* **22**, 1454-1466 (2016).
21. R. J. Blakemore *et al.*, Stability and conformation of the dimeric HIV-1 genomic RNA 5'UTR. *Biophysical journal* **120**, 4874-4890 (2021).
22. K. Lu *et al.*, NMR detection of structures in the HIV-1 5´-leader RNA that regulate genome packaging. *Science* **344**, 242-245 (2011).
23. T. E. M. Abbink, B. Berkhout, A novel long distance base-pairing interaction in Human Immunodeficiency Virus Type 1 RNA occludes the Gag start codon. *J. Biol. Chem.* **278**, 11601-11611 (2003).
24. J. D. Brown *et al.*, Structural basis for transcriptional start site control of HIV-1 RNA fate. *Science* **368**, 413-417 (2020).
